# Intelligent defense at the edge: a comprehensive survey of federated learning, TinyML and explainable AI for intrusion detection in IoT and IIoT ecosystems

**DOI:** 10.3389/frai.2026.1900815

**Published:** 2026-10-02

**Authors:** S. Sarath Kumar, M. Asha Jerlin

**Affiliations:** School of Computer Science and Engineering, Vellore Institute of Technology, Chennai, India

**Keywords:** federated learning, Industrial Internet of Things (IIoT), Internet of Things Security (IoT security), intrusion detection systems, lightweight machine learning, TinyML

## Abstract

The proliferation of Internet of Things (IoT) and Industrial Internet of Things (IIoT) technologies has fundamentally transformed contemporary computing infrastructures by interconnecting large heterogeneous devices, sensors, embedded systems, and cyber-physical platforms. These ecosystems support diverse applications including smart cities, intelligent transportation, industrial automation, medical surveillance, precision agriculture and home automation. Despite their operational advantages, the vast connectivity and inherent resource constraints of IoT devices significantly expand the cyberattack surface. Limited processing power, limited memory and restricted energy budgets render conventional cybersecurity solutions infeasible for IoT environments. Consequently, IoT systems have become prime targets for advanced threats, including malware, botnets, Distributed Denial of Service (DDoS) attacks, ransomware, insider attacks and data manipulation. Traditional Intrusion Detection Systems (IDS), designed for enterprise networks, rely on centralized data collection and computationally intensive machine learning models that are unsuitable for IoT deployments due to scalability limitations, communication overhead, latency constraints and privacy concerns. Recent research has therefore shifted toward lightweight, intelligent and distributed cybersecurity frameworks capable of providing robust protection under severe resource limitations. This survey comprehensively reviews recent advances in AI-based intrusion detection and malware analysis for IoT ecosystems, covering lightweight machine learning models (ML), deep learning (DL) architectures for edge deployment, federated learning (FL) based privacy-preserving detection, TinyML on-device intelligence, blockchain-based trust management, Explainable AI (XAI) driven transparent monitoring and adaptive cyber defense. A unified conceptual framework is proposed integrating lightweight detection, distributed collaborative learning, trust-based orchestration and adaptive defense.

## 1 Introduction

The Internet of Things is one of the most revolutionary technologies of the digital era. IoT ecosystems are made up of connected physical devices that have sensors, actuators, microcontrollers and communication interfaces to facilitate real-time data collection and automatic decision-making, forming a multi-layered architecture as illustrated in Figure 1.

**Figure 1:**
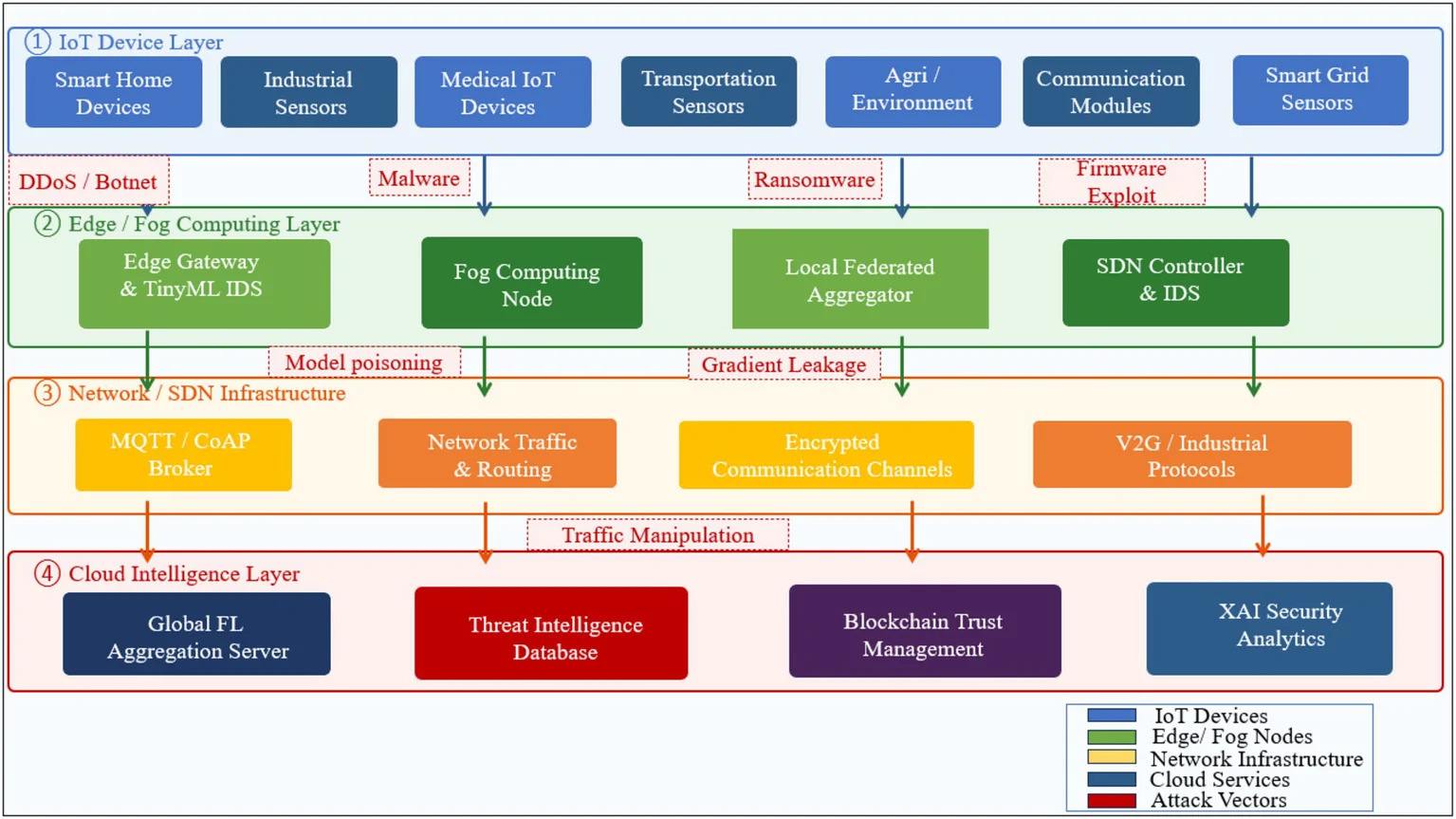
Multi-layer IoT ecosystem illustrating the relationships among IoT devices, Edge/Fog computing, network infrastructure, and cloud intelligence, together with representative cyberattack vectors and AI-enabled defense mechanisms for intelligent intrusion detection (created by the authors based on the reviewed literature).

Figure 1 presents the multi-layer IoT architecture considered throughout this review and illustrates the corresponding cyberattack surface across the IoT ecosystem. The device layer comprises heterogeneous endpoints, including smart home systems, industrial sensors, medical IoT devices, transportation infrastructure, agricultural monitoring systems, communication modules, and smart grid sensors. These devices continuously generate large volumes of distributed data while also representing common entry points for cyberattacks such as malware infections, botnet recruitment, ransomware, and firmware exploitation.

The Edge/Fog layer incorporates edge gateways, TinyML-enabled intrusion detection systems, fog computing nodes, federated learning aggregators, and SDN controllers to enable low-latency security analysis close to the data source. At the same time, collaborative and distributed learning environments introduce additional security risks, particularly model poisoning and gradient leakage attacks that target the learning process itself. Secure communication within the network layer is supported through protocols such as MQTT and CoAP, encrypted communication channels, and industrial networking technologies. Nevertheless, this layer remains vulnerable to traffic manipulation, routing attacks, and other network-oriented threats. At the cloud intelligence layer, global model aggregation, threat intelligence management, blockchain-based trust establishment, and Explainable AI (XAI) analytics collectively support coordinated intrusion detection across heterogeneous IoT deployments.

Unlike conventional IoT architecture diagrams that mainly emphasize communication hierarchies, Figure 1 integrates deployment layers, representative attack vectors, and AI-enabled defense mechanisms into a unified security perspective. It demonstrates how distinct cyber threats emerge across different layers of the IoT ecosystem while illustrating where technologies such as Lightweight AI, Federated Learning, TinyML, Blockchain, and Explainable AI can be deployed to mitigate those threats. As a result, the figure serves as the conceptual foundation for the remainder of this review by connecting architectural components with emerging intelligent intrusion detection technologies and highlighting their complementary roles in building secure, scalable, and privacy-preserving IoT ecosystems.

A network function virtualization system to detect malware in large IoT-based networks illustrates how cloud-hosted virtual functions can be used to monitor and safeguard masses of heterogeneous devices on a large scale (Guizani and Ghafoor, 2020). Future network security development in industrial IoT using AI-powered intrusion detection systems exemplifies the considerable entrenchment of intelligent security systems in manufacturing processes, energy systems and industrial control environments (Shahin et al., 2024). Malware detection systems powered by AI in industrial IoT settings further confirm the idea that complex industrial deployments need intelligent security measures to secure them (Smmarwar et al., 2023). In spite of such advantages, the massive use of IoT devices poses serious cybersecurity threats. IoT devices are often equipped with barebones computers and few security measures, making them easily exploitable (Sasikala and Janakiraman, 2023). Old firmware, poor authentication, or insecure communication protocols run on many machines (Ngo et al., 2020). Also, IoT devices are frequently used in geographically dispersed locations where direct physical monitoring is not feasible (Pandey et al., 2025).

IoT devices are becoming a common point of large-scale cyberattacks. Compromised IoT devices can be integrated into botnet networks capable of generating large amounts of malicious traffic (Nazir et al., 2023). Distributed Denial-of-Service (DDoS) attacks that shut down critical internet services have been conducted using these botnets (Lefoane et al., 2025). Moreover, cybercriminals exploit IoT vulnerabilities to obtain unauthorized access to industrial control systems, tamper with sensor data, or disrupt business operations (Hasan et al., 2022). Older cybersecurity methods geared towards enterprise networks do not fit the IoT environment. Centralized intrusion detection systems require continuous transmission of network traffic data to centralized servers where machine learning algorithms carry out detection and classification of anomalies (Ozdogan, 2024). Such centralized architectures in IoT ecosystems have exorbitant communication overhead and latency and increase privacy issues with sensitive data generated by devices (Abdulkareem et al., 2024). The field of machine learning and artificial intelligence has thus become an essential part of current IoT security systems (Shahin et al., 2024). Machine learning algorithms can identify malicious activity by processing vast amounts of network traffic data to determine patterns (Sharma and Bairwa, 2025). Deep learning architectures also improve detection through the automatic derivation of sophisticated features of network traffic streams, system logs and patterns of device behavior (Chaganti et al., 2022).

Nonetheless, traditional deep learning models are resource intensive and are not easily implemented on resource-limited IoT devices (Abualghanam et al., 2025). This constraint has encouraged the creation of lightweight machine learning frameworks tailored to edge computing applications (Kushwaha et al., 2025). These models focus on minimizing parameter complexity, effective feature selection and low inference latency at a tolerable level of detection accuracy (Reji et al., 2025). The next important innovation in the sphere of IoT security is the advent of federated learning, which allows jointly training models on distributed IoT devices without sending raw data to centralized servers (Rey et al., 2022). Instead, devices learn local models on their own data and only provide model updates to aggregation servers, maintaining data privacy and sharing collaborative intelligence (Alazab et al., 2023). Moreover, recent developments in TinyML technologies enable neural network models to be directly executed on microcontroller-size devices using methods such as quantization, pruning and parameter compression (Ramadan et al., 2025). These technologies allow intrusion detection features to be incorporated into the IoT devices themselves (Ficco et al., 2024). Besides these advancements, researchers are also studying blockchain-based trust management (Ali et al., 2024), explainable artificial intelligence for model transparency (Akshya et al., 2025) and adaptive moving target defense mechanisms that dynamically adjust system configurations to interfere with cyberattacks (Von Der Assen et al., n.d.).

### 1.1 Novelty and major contributions of this survey

This survey provides an integrated and holistic view of AI-based intrusion detection in the context of heterogeneous IoT and IIoT environments, a different approach from current survey papers that focus on individual paradigms like Federated Learning (FL), TinyML, Explainable AI (XAI) or Blockchain as standalone. The significant contributions of this survey can be summarized as follows:

- We propose a novel unified taxonomy that systematically categorizes AI-based Intrusion Detection approaches as Lightweight Machine Learning, Deep Learning, Federated Learning, TinyML, Blockchain-enabled Trust Management, Explainable AI, Dataset Engineering, Mathematical Foundations and Emerging Research Directions to give a structured and holistic organization of the current research scenario.
- We present a multidimensional comparative analysis framework that goes beyond traditional comparisons based on performance to consistently assess the current research based on extensive dimensions (datasets, attack categories, detection performance, computational complexity, communication overhead, energy consumption, privacy preservation, explainability, adversarial robustness, scalability, and deployment feasibility). This common evaluation framework will allow the comprehensive evaluation of AI-based intrusion detection systems for heterogeneous and resource-constrained IoT and IIoT environments.
- We define a common conceptual framework that enables the integration of Lightweight Machine Learning (LML) for efficient on-device inference tasks, TinyML (TL) for on-device low-power machine learning tasks, Federated Learning (FL) for collaborative analysis and model optimization in a privacy-preserving manner, Blockchain (BC) for decentralized trust management, and Explainable AI (XAI) for transparent and interpretable analysis and security decisions. The proposed framework goes beyond previous surveys that individually talk about these technologies and emphasizes their mutual complementarity, interactions, and deployment workflow for a scalable, trustworthy, energy-efficient and privacy-preserving intrusion detection.
- We deeply synthesize and review recent literature and systematically discuss the strengths and weaknesses of current intrusion detection (ID) approaches using AI together with their current practical deployment challenges and outstanding research gaps, going beyond mere descriptive summaries to pinpoint research trends and future development opportunities. We identify and define new research directions and open challenges such as Trustworthy Federated Intelligence, Energy Efficient TinyML, Adversarial Robust Learning, Explainable Cybersecurity, Quantum-resistant Security Mechanisms and Autonomous AI-driven Defense Architectures and offer a complete research roadmap for next-generation intelligent Intrusion Detection Systems.
- Table 1 presents a comparison of scope and the key contributions in the proposed survey with representative existing survey papers with a view to further highlighting the originality of this survey. The proposed survey is different from the previous reviews, which were mostly focused on specific research paradigms, because it proposes a unified taxonomy, a multidimensional comparison framework, a formalized conceptual framework and a comprehensive synthesis of the intrusion detection techniques used in the field of AI in heterogeneous IoT and IIoT environments.

**Table 1:** Comparison of existing survey papers and the proposed survey.

Feature	Existing surveys	This survey
Machine learning-based IDS	✓	✓
Deep learning-based IDS	Partial	✓
Federated learning	Some surveys	✓
TinyML	Few surveys	✓
Explainable AI (XAI)	Few surveys	✓
Blockchain-enabled trust	Few surveys	✓
Unified taxonomy	✗	✓
Multidimensional comparison framework	✗	✓
Unified conceptual framework	✗	✓
Dataset engineering	Partial	✓
Mathematical Foundations	✗	✓
Critical literature synthesis	Partial	✓
Research gap analysis	Partial	✓
Future research roadmap	Partial	✓
Covers both IoT and IIoT	Partial	✓

The proposed unified conceptual framework for AI-intrusion detection within heterogeneous IoT and IIoT ecosystems is shown in Figure 2. The framework starts from data acquisition from diverse IoT and IIoT devices, then collects data, preprocesses the data, and performs feature engineering to generate representative features for intrusion detection. Lightweight Machine Learning models and TinyML models can be deployed in an edge gateway to make efficient real-time inference with minimal computational overhead. In Federated Learning, only model parameters are shared to optimize the model collaboratively without compromising data privacy, resulting in better detection performance across the globe while maintaining data privacy. A key use of blockchain is to enable decentralized trust management by securely aggregating, authenticating and ensuring the integrity of model updates. Explainable AI improves transparency by providing explanations of the detection decisions and the highest value features that are causing the highest number of predictions. Regular feedback and updates to the models continue to make them more adaptive to new cyber threats. While the current survey covers the technologies separately, the proposed framework shows the complementary nature of the various technologies in a single intelligent intrusion detection architecture, thus enabling scalable, trustworthy, energy-efficient and privacy-preserving security for the complex heterogeneous IoT and IIoT environment.

**Figure 2:**
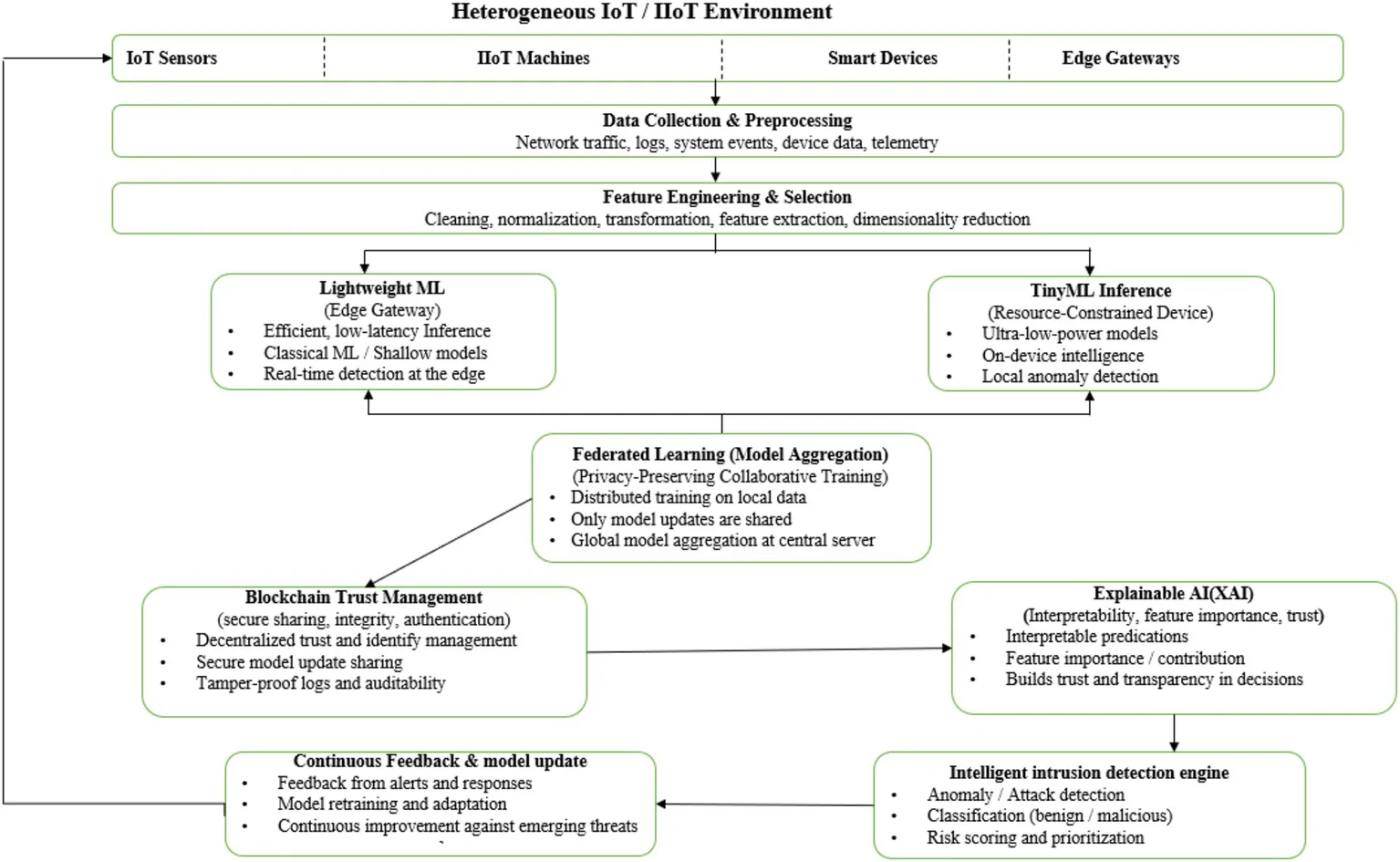
Proposed unified conceptual framework for AI-driven intrusion detection in heterogeneous IoT and IIoT ecosystems. The framework integrates Lightweight Machine Learning for low-latency edge inference, TinyML for resource-constrained on-device intelligence, Federated Learning for privacy-preserving collaborative model optimization, Blockchain for decentralized trust management, and Explainable AI(XAI) for transparent decision-making. Together, these complementary components enable scalable, energy-efficient, trustworthy, and explainable intrusion detection across heterogeneous IoT and IIoT environments (created by the authors based on the reviewed literature).

## 2 Methodology

Scopus is one of the largest multidisciplinary citation databases, indexing journals from IEEE, Elsevier, Springer, Wiley, Taylor & Francis, MDPI, ACM, and many other publishers. It provides broad coverage of high-quality peer-reviewed literature in engineering, computer science, and cybersecurity. Scopus supports advanced Boolean search queries, filtering by document type, language, publication year, subject area, and source type. Using a single comprehensive database improves the reproducibility of the search methodology by avoiding duplicate records across multiple databases.

### 2.1 Searching strategy

The literature search was conducted using the Scopus database due to its extensive coverage of peer-reviewed journals in computer science, artificial intelligence, cybersecurity, networking, and IoT research. The search employed the TITLE-ABS-KEY field with Boolean operators (AND, OR) to combine keywords related to IoT/IIoT, Intrusion Detection Systems (IDS), Artificial Intelligence (AI), and Blockchain/Trust Management. This search strategy ensured the comprehensive identification of relevant studies on intelligent intrusion detection for IoT and IIoT environments.

The following Boolean search query was used:

TITLE-ABS-KEY ((“Internet of Things” OR “IoT” OR “Industrial Internet of Things” OR “IIoT”) AND (“Intrusion Detection System” OR “IDS” OR “Intrusion Detection” OR “Network Intrusion Detection” OR “Anomaly Detection” OR “Network Anomaly Detection” OR “Malware Detection” OR “Malware Analysis” OR “Botnet Detection” OR “DDoS Detection” OR “Ransomware Detection” OR “Cyber Threat Detection” OR “Attack Detection”) AND (“Artificial Intelligence” OR “AI” OR “Machine Learning” OR “ML” OR “Deep Learning” OR “DL” OR “Lightweight Machine Learning” OR “TinyML” OR “Tiny Machine Learning” OR “Edge AI” OR “Edge Intelligence” OR “Federated Learning” OR “FL” OR “Distributed Learning” OR “Explainable Artificial Intelligence” OR “Explainable AI” OR “XAI”) AND (“Blockchain” OR “Trust Management” OR “Trust-Based” OR “Privacy Preserving” OR “Privacy-Preserving” OR “Secure Aggregation”)).

### 2.2 Search filters

To ensure the relevance and quality of the retrieved literature, several search filters were applied. Only peer-reviewed journal articles published between 2020 and 2026 and written in English were considered. The search was restricted to the TITLE-ABS-KEY field in the Scopus database, resulting in an initial retrieval of 2,231 records.

### 2.3 PRISMA chart

### 2.4 Inclusion and exclusion criteria

After determining the inclusion and exclusion criteria, the retrieved studies were screened based on these criteria (as shown in Table 2). Only peer-reviewed journal articles in English that focused on intrusion detection in IoT/IIoT scenario with the involvement of AI-based techniques were selected. To ensure methodological consistency and the quality of the final dataset, publications like conference papers, book chapters, editorials, duplicate records or studies not related to the review were excluded.

**Table 2:** Search filters and literature retrieval criteria.

Parameter	Selection
Database	Scopus
Search field	TITLE-ABS-KEY
Publication period	2020–2026 (replace with your actual years)
Document type	Journal Articles
Language	English
Source type	Peer-reviewed Journals
Initial records retrieved	2,231

### 2.5 Study classification

The articles were classified in a systematic way after the final selection of 114 studies was made, for the comparative study. Finally, the selected studies were categorized into six analytical dimensions: (i) intrusion detection paradigms (signature-, anomaly-, and hybrid-based IDS), (ii) artificial intelligence techniques (machine learning, deep learning, federated learning, TinyML, and explainable AI), (iii) deployment architecture (cloud, edge, fog, and federated environments), (iv) security enhancement mechanisms (blockchain, trust management, and privacy-preserving approaches), (v) datasets and evaluation metrics, and (vi) research challenges and future directions. This classification system is used throughout the rest of this review for the comparative discussion.

*PRISMA Summary*: The literature search was performed by applying a Boolean search using a predefined search strategy TITLE-ABS-KEY to the Scopus database, which resulted in 2,231 records from the literature. At the identification stage, 186 duplicates, 42 publications in languages other than English and 23 retracted or invalid records were identified and excluded, and 1,980 records were submitted to the title and abstract screening stage. In the Screening stage, 1,416 documents were excluded due to their irrelevance to the theme of this review; these included studies that did not relate to IoT/IIoT intrusion detection (*n* = 274), studies that did not use AI/ML techniques (*n* = 198), studies that were not related to network security (*n* = 76), and studies from other research domains (*n* = 52), along with other irrelevant publications (*n* = 816). The remaining 564 full-text articles were assessed for eligibility. Following careful review, 450 papers were eventually removed because they lacked sufficient technical details (*n* = 114), were conference posters or abstracts (*n* = 96), did not focus on IoT/IDS security (*n* = 87), were not relevant to the research questions (*n* = 78), were low quality or not peer-reviewed (*n* = 46), and were older and less relevant (*n* = 29). After the quality-based assessment, 114 peer-reviewed journal articles were selected and were utilized in the qualitative synthesis and comprehensive analysis of this review.

### 2.6 Experimental settings analysis of the reviewed studies

To provide a comprehensive comparison of the reviewed literature, the experimental configurations reported in the selected 114 studies were systematically analysed. The extracted information included the datasets employed, representative intrusion detection models, commonly reported parameter settings, hardware and software environments, evaluation metrics, and attack scenarios. Since this survey does not conduct original experiments, the table summarizes the experimental practices adopted across the reviewed studies rather than presenting a unified experimental setup. This analysis enables readers to understand the typical implementation environments and evaluation methodologies used for AI-driven intrusion detection in IoT and IIoT systems.

According to the analysis, CICIDS2017, ToN-IoT, CICIoT2023 and BoT-IoT are the most used reference datasets to assess the performance of IoT intrusion detection systems. Typical hyperparameters for deep learning studies include learning rates of approximately 0.001, batch sizes of 32 to 64, and local epochs. Federated learning studies also report on communication rounds and clients that participate. The popular implementations use TensorFlow, PyTorch or Scikit-learn and are tested on an edge platform like Jetson Nano and Raspberry Pi or workstations with GPUs. These notes reflect the variety of experimental setups found among the studies examined, and serve as a reference for others to use in the future (Figure 3).

**Figure 3:**
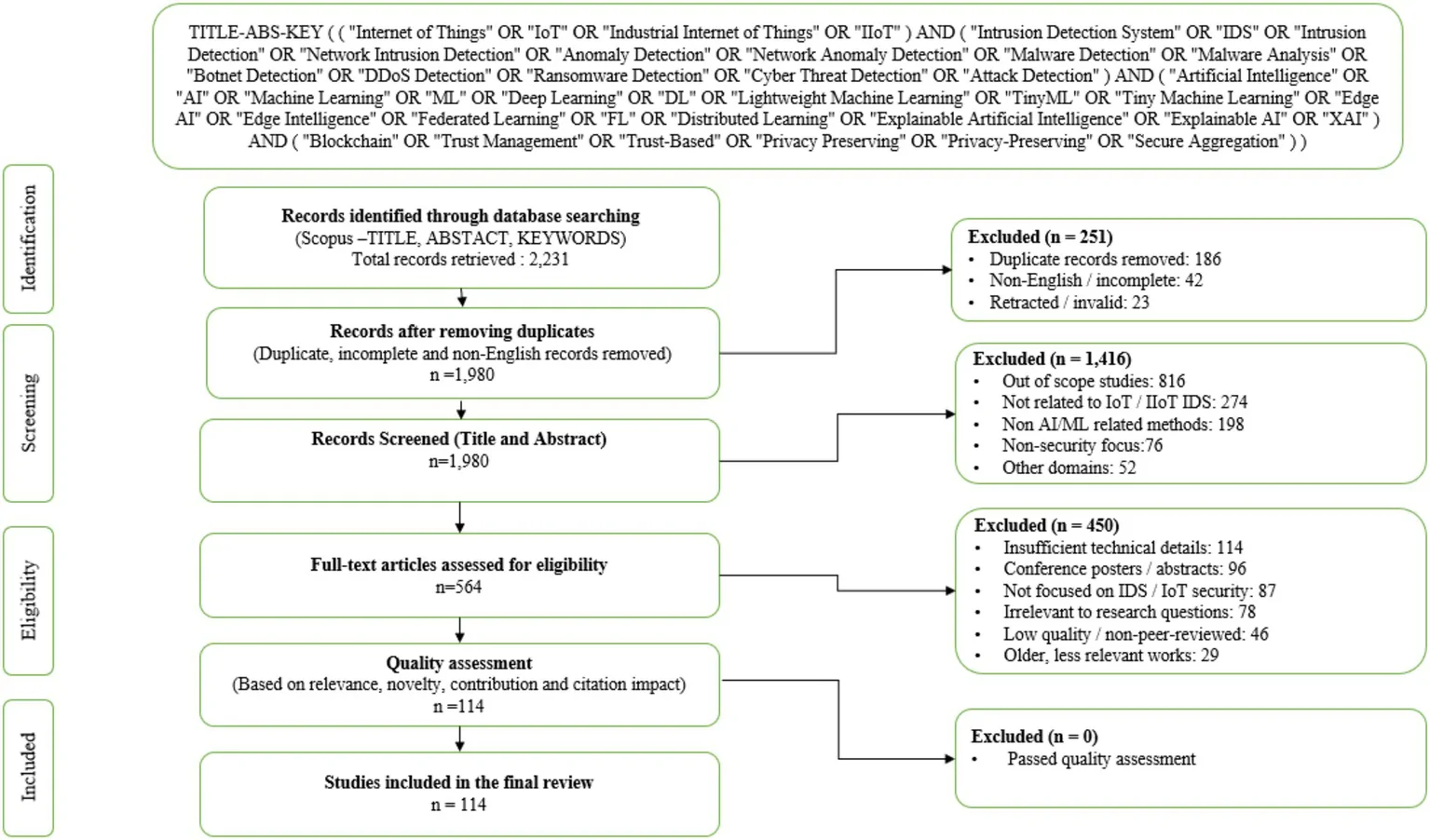
PRISMA flow diagram of study selection with search strategy, inclusion and exclusion criteria.

## 3 IOT threat landscape and attack taxonomy

For the purposes of this review, the threat taxonomy has been implemented and categorized the IoT cyber threats based on the layers they are targeting, the most representative attack techniques based on them and resulting operational impacts, as presented in Figure 4. The taxonomy identifies three main types of attacks—network level, device level, and data level attacks. While the former fall in the category of network-level attacks, the latter are device-level attacks that exploit vulnerabilities in the firmware, operating system and IoT applications that devices depend on. Data-oriented attacks aim to alter the data gathered by sensors and machines, as well as confidential information, to infiltrate the integrity and confidentiality of intelligent IoT services.

**Figure 4:**
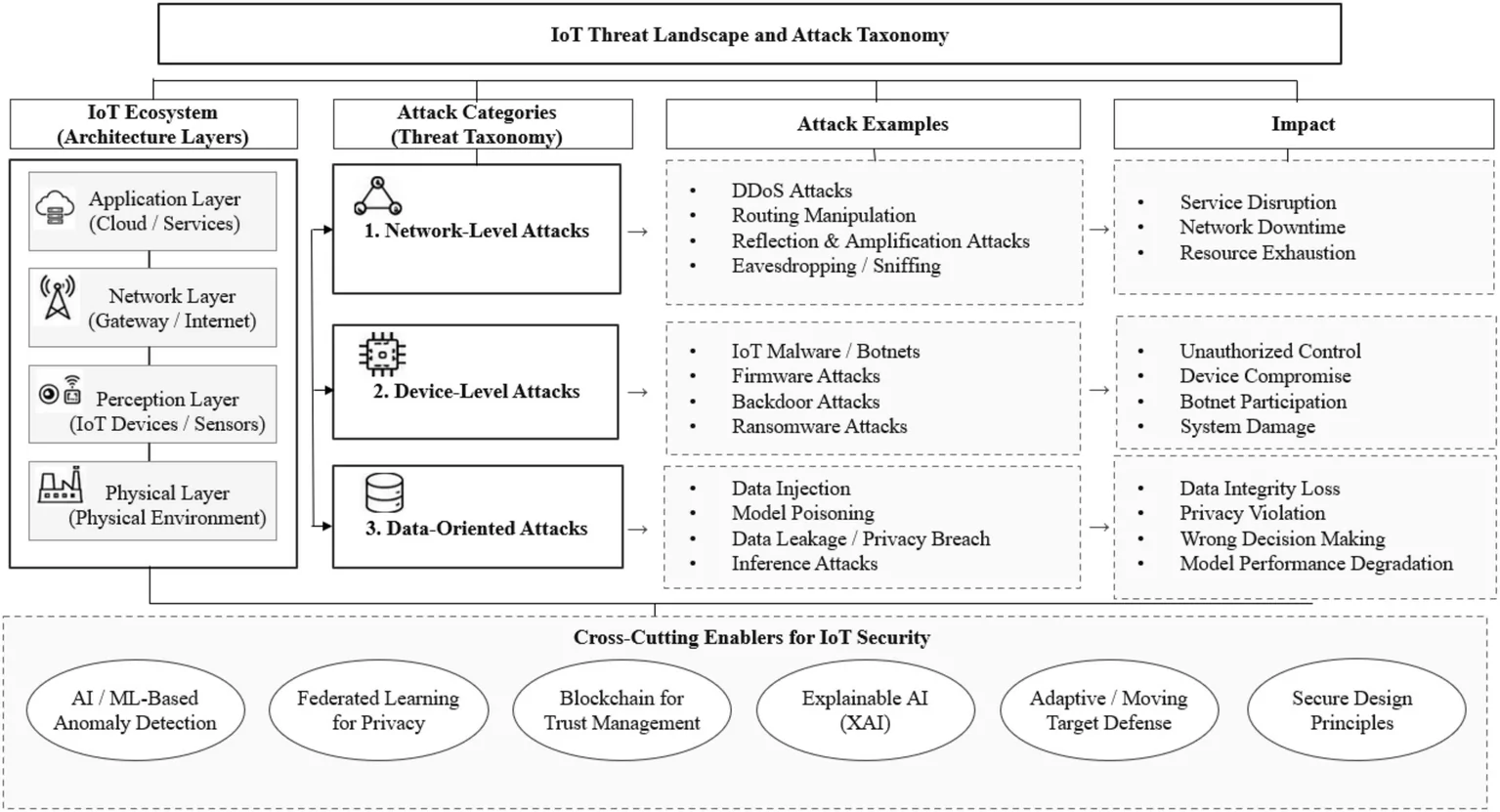
IoT threat taxonomy illustrating the relationship between IoT architecture layers, major attack categories, representative attack techniques, their operational impacts, and cross-cutting AI-enabled security mechanisms (created by the authors based on the reviewed literature).

Figure 4 depicts not only the categorization of attacks, but also the connection between attack categories and the development of new AI-based security mechanisms. The figure shows that, instead of handling attacks separately, Lightweight AI, Federated Learning, Blockchain, Explainable AI, Adaptive Moving Target Defense and the concept of secure-by-design work together across various layers of the architecture to detect, mitigate, and respond to attacks. The integrated taxonomy serves as an analytical lens to the next sections, each of which investigates a security paradigm in more detail (Tables 3, 4).

**Table 3:** Inclusion and exclusion criteria.

**Inclusion criteria**	**Exclusion criteria**
Peer-reviewed journal articles	Conference papers
Published between 2020–2026	Book chapters
English-language publications	Editorials and notes
Studies focusing on IoT/IIoT intrusion detection	Duplicate records
AI-, ML-, DL-, Federated Learning-, TinyML-, XAI-, or blockchain-based IDS studies	Non-English publications
Studies reporting architectures, datasets, evaluation metrics, or experimental results	Studies unrelated to IoT/IIoT or IDS
Relevant to IoT and IIoT cybersecurity	Papers with insufficient technical details or incomplete evaluations

**Table 4:** Summary of experimental settings reported across the reviewed studies.

Experimental component	Summary from the 114 reviewed papers
Datasets	ToN-IoT, CICIDS2017, CICIoT2023, BoT-IoT, UNSW-NB15, MQTT-IoT-IDS2020, WSN-DS and other IIoT datasets
Representative models	Random Forest, SVM, XGBoost, CNN, LSTM, CNN-LSTM, GRU, FedAvg, FedProx, TinyML CNN, Blockchain-assisted IDS, XAI-based IDS
Typical parameter settings	Learning rate (0.0001–0.01), batch size (16–128), epochs (20–200), Adam/SGD optimizer, local epochs (1–10), communication rounds (20–200), clients (5–100), INT8 quantization for TinyML
Hardware platforms	Intel Core i5/i7/i9, AMD Ryzen, NVIDIA GTX/RTX GPUs, Jetson Nano/Xavier, Raspberry Pi, ESP32, STM32, Edge gateways
Software frameworks	Python, TensorFlow, TensorFlow Lite, TensorFlow Federated, PyTorch, Scikit-learn, Keras, Flower, Hyperledger Fabric, Ethereum
Evaluation metrics	Accuracy, Precision, Recall, F1-score, ROC-AUC, MCC, Detection Rate, False Positive Rate, Latency, Energy Consumption
Attack scenarios	DDoS, DoS, Botnet, Malware, Ransomware, Probe, U2R, R2L, Insider Attack, Model Poisoning, Gradient Leakage

The threat landscape of IoT settings is marked by various attack vectors that may target various levels of the system architecture (Sasikala and Janakiraman, 2023). These threats have weaknesses in the firmware of the device, communication protocol, network infrastructure and application software. Knowledge of the taxonomy of IoT threats is critical in developing effective intrusion detection and mitigation strategies (Almobaideen et al., 2025). Three main groups of IoT cyber threats can be identified: network-level attacks, device-level attacks and data-oriented attacks, as illustrated in Figure 4. These categories denote different types of attacks on various elements of the IoT ecosystem (Bala and Behal, 2024). A detailed classification of IoT cyber threats, including attack types, target layers, impacts and techniques, is presented in Table 5.

**Table 5:** IoT cyber-threat taxonomy.

Attack category	Attack type	Target layer	Primary impact	Example techniques	Key refs
Network-level	DDoS/flooding	Network/transport	Service disruption	Botnet orchestration, amplification	Lefoane et al. (2025); Bala and Behal (2024); Nawaz et al. (2025)
Network-level	Routing manipulation	Network	Traffic interception	Protocol spoofing, sinkhole	Ngo et al. (2020); Pandey et al. (2025)
Network-level	Reflection/amplification	Network	Bandwidth exhaustion	DNS amplification, NTP reflection	Pandey et al. (2025); Nazir et al. (2023)
Device-level	IoT Malware	Device/OS	Device compromise	Persistence, botnet recruitment	Lefoane et al. (2025); Hasan et al. (2022) Ozdogan (2024)
Device-level	Firmware exploitation	Firmware	Persistent backdoor	Unsigned firmware, buffer overflow	Ngo et al. (2020); Abdulkareem et al. (2024)
Device-level	Ransomware	Application	Operational Disruption	Encryption, Function lockout	Sharma and Bairwa (2025); Chaganti et al. (2022)
Device-level	Backdoor injection	Firmware/OS	Unauthorized access	Remote shell, hidden credentials	Abdulkareem et al. (2024)
Data-oriented	Data injection	Application/sensing	Sensor manipulation	False data injection, replay	Guizani and Ghafoor (2020); Shahin et al. (2024)
Data-oriented	Model poisoning	ML Model	Detection Evasion	Label flipping, Gradient manipulation	Smmarwar et al. (2023)
Data-oriented	Gradient leakage	Federated learning	Privacy breach	Gradient inversion, Inference attack	Sasikala and Janakiraman (2023); Smmarwar et al. (2023)

### 3.1 Network-level attacks

At the network level, attacks can be directed at communication channels among IoT devices, gateways and cloud servers that include disrupting communication, altering data sent through them and using network protocols to infiltrate network devices they have unauthorized access. One of the most prevalent IoT-related network-level threats is distributed denial-of-service attacks. Compromised IoT devices are exploited to produce massive traffic to target servers or network infrastructure (Bala and Behal, 2024).

IoT botnets are characterized by a large-scale, which significantly increases the potential effects of such attacks (Kumar et al., 2022). To effectively identify DDoS attacks within IoT networks, researchers have suggested various lightweight machine learning frameworks (Nawaz et al., 2025). Federated learning-based wireless sensor network detection systems also tackle DDoS threats within smart cities (Devi et al., 2025). Another type of network-level attack is routing manipulation attacks (Ngo et al., 2020). Reflection and amplification attacks take advantage of network services that, in response to small requests, send out large responses (Nazir et al., 2023).

### 3.2 Device-level attacks

Attacks at the device level are aimed at the inner workings of IoT devices and are carried out using the vulnerabilities of the device firmware, operating systems, or application software to obtain unauthorized control. One of the most serious threats to devices is IoT malware. Attacks on IoT devices are frequently persistent, stealthy and scalable (Sasikala and Janakiraman, 2023). After being infected, a device can be involved in botnet activities, scan networks and find other vulnerable devices or receive remote instructions from command-and-control servers (Alhamdi et al., 2025). An especially threatening type of malware in industrial IoT systems is backdoor malware (Khan et al., 2024). Firmware attacks involve installing malicious code or bypassing security controls through firmware vulnerabilities (Ngo et al., 2020). Another emerging threat is the use of ransomware attacks on IoT systems (Mofidi et al., 2024). A thorough analysis of ransomware attacks, detection and countermeasures emphasizes the increasing intensity of this threat (Yan and Khoei, 2025).

### 3.3 Data-oriented attacks

Data attacks are aimed at manipulating or obtaining sensitive data on the IoT systems and, in most cases, on machine learning models that are utilized to detect anomalies and make decisions. In data injection attacks, an attacker adds rogue data to IoT data streams to control system behavior (Guizani and Ghafoor, 2020). Permission-level risk profiling has been suggested as an approach to identify anomalies due to unauthorized data access patterns (Shahin et al., 2024). Model poisoning attacks are specifically applied to machine learning models involved in intrusion detection systems (Smmarwar et al., 2023). An example of real-world privacy-saving Android malware detection by federated machine learning shows that federated participants need to exercise cautious model updating behavior to avoid the eventual accidental leaking of sensitive device data (Sasikala and Janakiraman, 2023).

## 4 Evolution of intrusion detection systems in IOT

Intrusion detection systems are essential in detecting malicious activities in the IoT networks (Abualghanam et al., 2025). Intrusion detection technologies have, over the years, developed beyond simple rule-based systems to complex artificial intelligence-based systems that can identify advanced cyber threats (Kushwaha et al., 2025). Figure 5 summarizes the generational progression of intrusion detection systems, highlighting key techniques, strengths and limitations across different phases.

**Figure 5:**
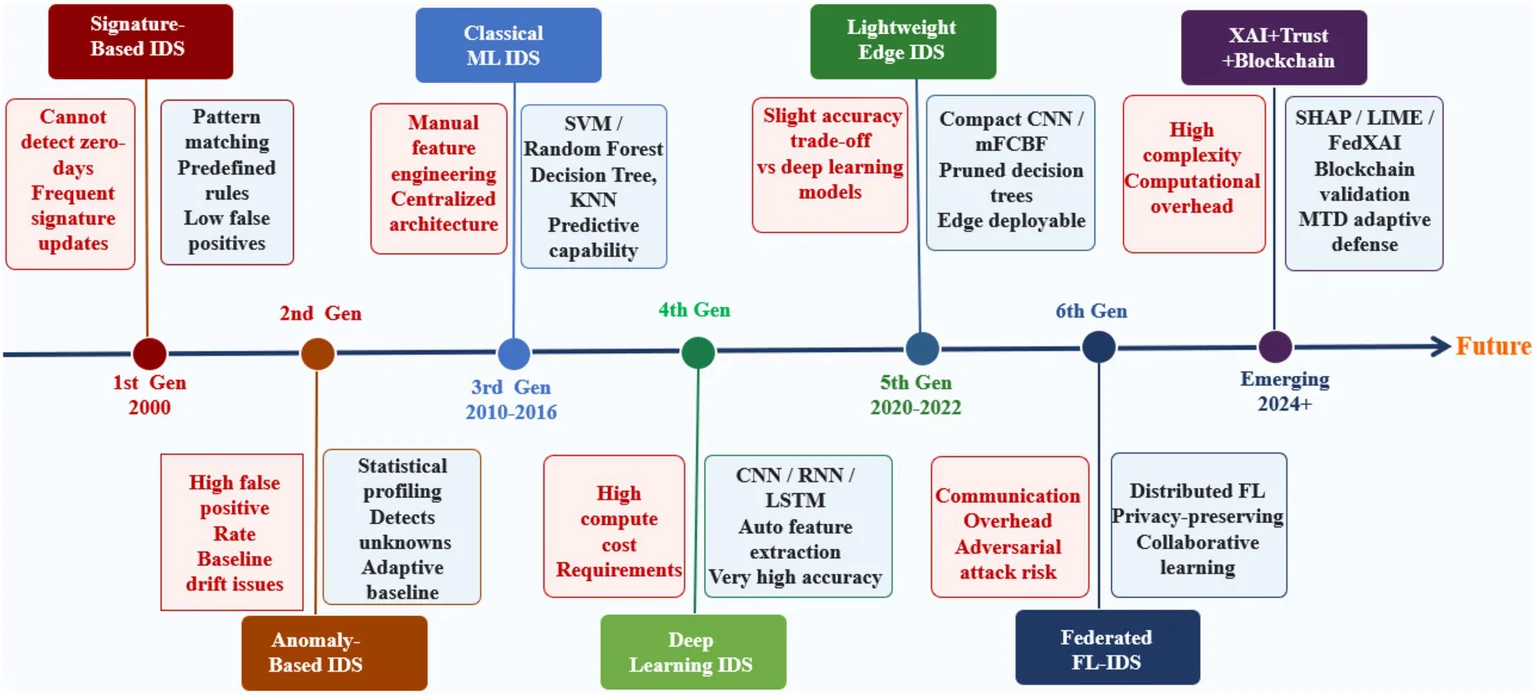
Evolution of IoT intrusion detection systems from signature-based detection to AI-driven, federated, explainable, and trust-aware architectures, highlighting the major techniques, strengths, limitations, and deployment characteristics across successive generations (created by the authors based on the reviewed literature).

Figure 5 shows the evolution of intrusion detection systems (IDS) for the IoT environment, ranging from simple signature-based IDSs to intelligent, distributed, and explainable security systems. The evolution confirms that cyber threats within the IoT landscape are growing in complexity, and so must be the mechanisms in place to detect these threats. Early generations used pre-defined signatures and statistical anomaly detection, but later generations adapted classical machine learning, deep learning, lightweight edge intelligence, federated learning, explainable AI and blockchain for greater accuracy, scalability, privacy protection, and deployment feasibility.

Figure 5 shows how each generation overcomes the constraints imposed by the previous generation, rather than being a separate technological advancement. Signature-based systems work well against known attacks but are not useful in detecting zero-day attacks. Anomaly (departure) detection is more accurate at the cost of increased false positives. Predictive capability can be improved by classical machine learning, but comes with a significant amount of manual feature engineering, while deep learning simplifies the process of feature extraction but requires more computational power. Recent decentralized lightweight, and explainable IDS architectures mitigate these issues by enabling resource-constrained edge devices, privacy-preserving collaborative learning, and transparent security decision making. The conceptual model set out in Figure 5 is thus used as the basis for the rest of the present review of the technological evolution.

The first intrusion detection systems were based on signature-based detection. Hybrid intrusion detection methods based on signatures and anomalies have been used to protect IoT and vehicle-to-grid V2G communications (Reji et al., 2025). Intrusion detection systems based on anomaly appeared as an alternative that is modelled by normal system behavior and detects anomalies that can be an indication of malicious activity (Rey et al., 2022). Although this method is effective at detecting unfamiliar attacks, it has a high false-positive rate because of the dynamic characteristics of the IoT traffic patterns (Alazab et al., 2023). A detailed comparison of intrusion detection system generations, including their techniques, strengths and limitations, is presented in Table 6.

**Table 6:** Evolution of intrusion detection systems in IoT.

Generation	Detection approach	Core technique	Strengths	Limitations	Refs
1st Gen	Signature-Based	Pattern Matching	High accuracy on known attacks, Low FP	Cannot detect zero-days, Requires frequent updates	Abualghanam et al. (2025); Kumar et al. (2022)
2nd Gen	Anomaly-Based	Statistical Profiling	Detects unknown attacks, Adaptive	High false positive rate, Baseline drift	Alazab et al. (2023); Rey et al. (2022)
3rd Gen	Classical ML-Based	SVM, Random Forest, Decision Tree	Improved accuracy, Predictive capability	Manual feature engineering, Centralized	Shahin et al. (2024); Ozdogan (2024)
4th Gen	Deep Learning-Based	CNN, RNN, LSTM, Hybrid	Automated feature extraction, High accuracy	High compute cost, large data needed	Hasan et al. (2022); Akshya et al. (2025); Ali et al. (2024)
5th Gen	Lightweight Edge-Based	Compact CNN, mFCBF, Pruned Trees	Edge deployable, Low latency, Low energy	Slightly lower accuracy vs. deep models	Nawaz et al. (2025); Devi et al. (2025); Von Der Assen et al. (n.d.); Alhamdi et al. (2025)
6th Gen	Federated and distributed	FL, TinyML, Secure Aggregation	Privacy-preserving, Collaborative	Communication overhead, Adversarial risk	Khan et al. (2024); Khraisat et al. (2025); Bhatia et al. (n.d.); Mofidi et al. (2024)
Emerging	XAI + Trust + Blockchain	SHAP, LIME, Smart Contracts, MTD	Transparent, Trustworthy, Adaptive	Computational overhead, Complexity	Yan and Khoei (2025); Al Abdulwahid (2025); Singh and Siddalingaiah (2025); Eslamnejad et al. (2025)

New machine learning algorithms have added predictive features to intrusion detection systems. Algorithms like Support Vector Machines, Random Forests and Decision Trees can be used to analyze statistical characteristics of network traffic flows (Abualghanam et al., 2025). An in-depth study of machine learning algorithms in IoT IDS proves the flexibility of these algorithms in various deployment situations (Ramadan et al., 2025). Intrusion detection has also been enhanced by deep learning architectures (Hasan et al., 2022). Convolutional neural networks (CNN) have been shown to be quite useful in detecting spatial patterns in network traffic data (Ficco et al., 2024). Temporal dependencies are dealt with in recurrent neural networks and hybrid CNN-RNN models (Ali et al., 2024). Although deep learning models have better accuracy, they need a lot of computing power and cannot be used in environments with limited resources, such as IoT (Akshya et al., 2025). Researchers, in turn, have been working on lightweight deep learning models that can be used effectively on edge devices (Von Der Assen et al., n.d.). Federated learning framework integration is another important development (Almobaideen et al., 2025). This development is recorded in a thorough survey of privacy-preserving federated learning in the context of intrusion detection in IoT (Bala and Behal, 2024).

These emerging paradigms are examined individually in Sections 9–11 to illustrate how blockchain, trust-aware learning, and explainable AI collectively enhance the robustness, transparency, and reliability of modern IoT intrusion detection systems.

## 5 Lightweight machine learning architectures for IoT intrusion detection

As shown in Figure 6, the complete lightweight machine learning solution employed for IoT intrusion detection is the following: Raw network traffic acquisition, data preprocessing, feature selection, dimensionality reduction, lightweight classification, and finally, attack detection. The pipeline illustrates the application of preprocessing and feature selection methods, which reduce the dimensionality of the data to a smaller set of features, before being classified by simple classifiers with low computational cost. This design has the advantages of low resource consumption, fast inference speed, and small computation output, and at the same time guarantees that the intrusion detection performance of the resource-limited IoT equipment is acceptable.

**Figure 6:**
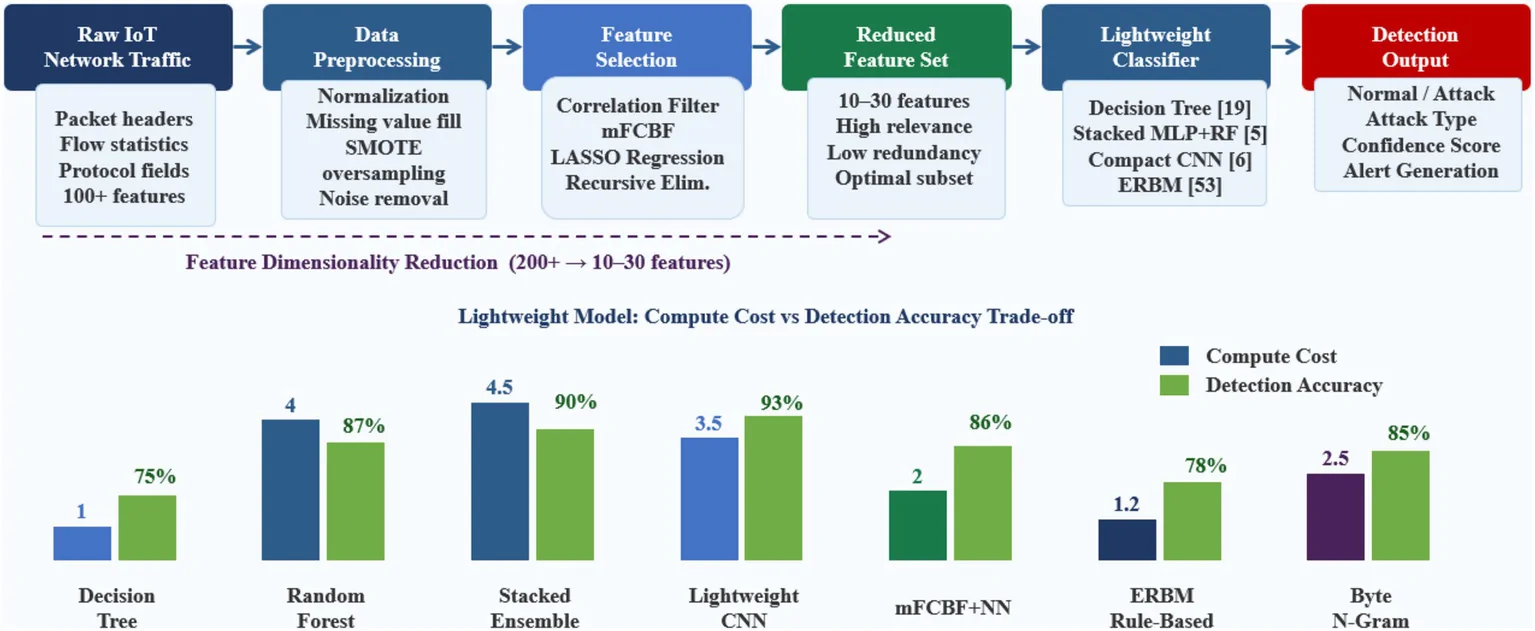
Lightweight machine learning pipeline for IoT intrusion detection illustrating feature preprocessing, dimensionality reduction, lightweight classifier selection, and the trade-off between computational cost and detection accuracy (created by the authors based on the reviewed literature).

Figure 6 is intended to demonstrate the processing workflow as well as the underlying tension between the degrees of efficiency and accuracy in developing lightweight IoT IDSs. The comparison of representative lightweight classifiers shows that choosing the proper classifier can not only rely on prediction accuracy, but also on deployment factors such as memory, processing latency, and energy consumption. As a result, feature engineering, dimensionality reduction and lightweight classifier optimization become crucial issues when trying to enable real-time intrusion detection on edge and embedded IoT devices. The conceptual pipeline is the basis for the light weight machine learning techniques throughout this section.

The comparative performance demonstrated in the bottom of Figure 6, suggests that the lightweight CNN-based and mFCBF-based models have a better trade-off between computation and detection accuracy compared to traditional ensemble models, which make them well suited for use in resource-limited IoT systems. The concept of a pipeline is the foundation for the light-weight machine learning methods described in this section.

The high rate of Internet of Things ecosystem growth has increased the importance of effective cybersecurity systems that can perform under stringent computational requirements (Nawaz et al., 2025). These constraints do not allow immediate implementation of computationally intensive security models that are typically employed in enterprise networks (Abualghanam et al., 2025). A comparative analysis of lightweight machine learning models, including their feature selection methods, computational costs and detection performance, is presented in Table 7.

**Table 7:** Comparison of lightweight machine learning models for IoT intrusion detection.

Model/method	Category	Feature selection	Compute cost	Edge feasibility	Detection accuracy	Refs
Decision Tree (Pruned)	Classical ML	Correlation-Based	Very Low	Very High	Moderate	Abualghanam et al. (2025); Ciaramella et al. (2026)
Random Forest	Ensemble	Feature Importance	Moderate	High	High	Baimukhanov et al. (2025); Khraisat et al. (2025)
Stacked Ensemble (MLP+RF)	Ensemble	Recursive Elimination	Moderate	Moderate	High	Baimukhanov et al. (2025)
mFCBF + Lightweight NN	Filter + NN	mFCBF	Low	Very High	Moderate–High	Devi et al. (2025)
Lightweight CNN	Deep Learning	Automatic (Depth-wise)	Low–Moderate	Moderate–High	Very High	Bhatia et al. (n.d.); Villafranca and Cano (2025)
ERBM Rule-Based Model	Rule-Based ML	Domain Rules	Very Low	Very High	Moderate	Ciaramella et al. (2026)
Byte N-Gram Classifier	Static Analysis	N-Gram Extraction	Low	High	Moderate–High	Kalakoti et al. (2025)
XGBoost/gradient boost	Ensemble	LASSO/Embedded	Moderate	Moderate	High	Alazab et al. (2023); Nazir et al. (2023)

Edge/Fog Computing Layer: Lightweight machine learning model deployed to detect intrusions in real time close to where data is produced, using Edge Gateways and TinyML supported IDS (Figure 1). This deployment minimizes the delay and computation time of communication and can rapidly identify attacks from diverse IoT devices.

The computational restrictions of resource constrained IoT edge devices fall to lightweight machine learning techniques, which are mainly used to detect network level attacks like DDoS, routing manipulation, routing botnet traffic, as shown in Figure 4.

Lightweight models focus on lesser computational complexity and yet possess effective detection capabilities (Devi et al., 2025). The main design goal is to achieve minimal training time, minimal memory usage and minimal latency inference with minimal intellectual loss in classification (Ciaramella et al., 2026). To balance this, feature selection strategies, model architectures and data preprocessing pipelines have to be optimized (Alhamdi et al., 2025). Forming a lightweight detection pipeline as illustrated in Figure 6.

### 5.1 Feature engineering and dimensionality reduction

Feature engineering plays a crucial role in lightweight intrusion detection systems. Hundreds of attributes are usually present in the raw IoT network traffic, many of which are redundant or irrelevant to detecting attacks (Devi et al., 2025). Learning-based android malware detection using feature-driven static analysis shows how a wise choice of features can save a lot of computational power (Alnasser et al., 2025).

Feature engineering and dimensionality reduction form the backbone of the lightweight detection pipeline, as shown in Figure 6, and help to identify a set of select traffic attributes while minimizing computation complexity and retaining discriminative information.

The feature selection methods are intended to determine the most informative features (Alazab et al., 2023). Modified Fast Correlation-Based Filter (mFCBF) feature selection techniques have been used to create lightweight intrusion detection systems for IoT networks (Devi et al., 2025). Recursive feature elimination recursively goes through the features and eliminates the least significant features according to model performance measures (Ciaramella et al., 2026). The resulting dimensionality reduction is highly effective in enhancing the efficiency of the training process and decreases the amount of computation required of the IoT devices (Baimukhanov et al., 2025).

### 5.2 Decision tree-based lightweight detection models

Decision tree algorithms are among the most popular lightweight machine learning methods of detecting IoT intrusions (Abualghanam et al., 2025). Their simplicity of computation and interpretability make them popular (Ciaramella et al., 2026). In contrast to deep learning models, decision trees do not need large training sets and only use a few computational resources to perform inference (Alazab et al., 2023). A machine learning-based malware detection method, which improves cloud privacy protection, shows how the tree-based classifiers can be adapted to limited environments (Sun and Wang, 2025). Using wide residual networks as a method to detect obfuscated malware in IoT devices further expands tree-based paradigms (Selvam et al., 2025).

### 5.3 Ensemble learning for lightweight intrusion detection

Ensemble learning algorithms are a combination of several classifiers to enhance their detection accuracy and resilience. A stacked ensemble model combines many base classifiers (Esmaeilyfard et al., 2025). A lean and mean model of botnet detection in IoT based on stacked ensemble learning illustrates that a multilayer perceptron with a random forest classifier model would offer better detection results (Baimukhanov et al., 2025). A feature-learning-based malware analysis system also shows that the combination of multiple feature extractors is beneficial (Nedungadi et al., 2025). Nevertheless, ensemble learning presents some extra computational complexity as well. Lightweight ensemble models will often have fewer base learners and can optimize feature selection (Khraisat et al., 2025).

### 5.4 Lightweight convolutional neural networks

The use of convolutional neural networks has become popular in the study of intrusion detection because they have the capacity to automatically identify hierarchical features of the input data (Bhatia et al., n.d.). Lightweight CNNs are architectures made to execute on resource-constrained systems that minimize the number of layers in the network and use smaller convolutional filters, with depth-wise separable convolutions further decreasing the amount of computation (Villafranca and Cano, 2025).

A small CNN-based malware classification algorithm to detect malware in software shows that CNNs can be used to detect malware activities with minimum parameter overhead (Bhatia et al., n.d.). CNN-based detection on embedded devices with IoT malware classification based on lightweight neural networks further confirms the viability of CNN (Vyas et al., 2024).

Another lightweight approach to malware classification on IoT devices through machine learning and the use of byte N-grams to classify malware is another method (Kalakoti et al., 2025). The relevance of these architectures to residential IoT settings is shown by a privacy-friendly lightweight IDS to defend against smart home botnets (Mehmmod et al., 2025). The new deep-boosted CNN and ensemble learning-based IoT malware detection system has a high detection accuracy (Khraisat et al., 2025). GuardDroid is a lightweight and transparent malware-detection method used to protect Android IoT devices that supplements CNN-based detection with the use of the static analysis method (Kharnotia et al., 2026). The effectiveness of machine learning in detecting botnet attacks with IoT Security improves proves that lightweight classifiers are competitive in detecting botnet attacks on real-world datasets (Esmaeilyfard et al., 2025). The direction to lightweight hybrid deep learning methods for malware detection improvement in IoT-based systems proves that small neural networks can be used to reach the state-of-the-art performance (Baawi et al., 2025). The problem of scalability is overcome by ultra-lightweight and safe intrusion detection systems of massive IoT networks (Alsekait et al., 2024).

## 6 Deep learning architectures for IoT intrusion detection

Deep learning models have enhanced intrusion detecting systems by a great deal (Hasan et al., 2022). Deep learning architectures in the context of IoT security studies are recommended to be customized to achieve a balance between detection accuracy and computational efficiency (Akshya et al., 2025). A comparative analysis of deep learning architectures for IoT intrusion detection, including their input types, computational requirements and deployment feasibility, is presented in Table 8.

**Table 8:** Deep learning architecture comparison for IoT security.

Architecture	Input type	Spatial analysis	Temporal analysis	Compute cost	Edge deployable	Refs
Standard CNN	Image/feature matrix	High	Low	Moderate	Moderate	Hasan et al. (2022); Ficco et al. (2024)
Lightweight CNN	Feature matrix/packet	High	Low	Low	High	Bhatia et al. (n.d.); Villafranca and Cano (2025); Vyas et al. (2024)
LSTM/GRU	Sequential traffic flows	Low	Very high	High	Low	Ali et al. (2024)
Hybrid CNN-LSTM	Network traffic	High	High	High	Moderate	Ali et al. (2024); Von Der Assen et al. (n.d.)
Variational autoencoder	Network traffic	Moderate	Low	Moderate	Moderate	Bonsu and Akekudaga (2025); Wajahat et al. (2024)
GAN/CTGAN (Augmentation)	Tabular/traffic	Moderate	Low	High	Low	Wajahat et al. (2024)
Image-based DNN	Binary visualization	High	Low	Moderate	Moderate	Ficco et al. (2024); Zhukabayeva et al. (2024)
Transformer/BERT-based	Network logs/text	High	High	Very high	Low	Mittal and Rajvanshi (n.d.)

### 6.1 Recurrent neural networks for sequential traffic analysis

Data in network traffic frequently has temporal interactions that are not well represented by static features. This problem is solved by recurrent neural networks (RNN) (Ali et al., 2024). There is a wide usage of Long Short-Term Memory (LSTM) networks in intrusion detection systems. The LSTM networks add memory cells and gating mechanisms that allow the model to remember valuable information across long sequences (Ali et al., 2024). Within a botnet-communication situation, there can be a series of behavior to be exhibited by an infected machine. An IoT botnet detection system using extreme learning machines illustrates the effectiveness of modelling sequential behavior patterns (Alzubi et al., 2025). Early detection of IoT botnets with machine learning and network-edge traffic also confirms the usefulness of temporal analysis (Khan et al., 2023). Even though LSTM models offer robust sequential analysis features, they are computationally expensive, making them difficult to use on low-power IoT devices (Chen J. et al., 2025). Consequently, LSTM-based intrusion detection systems are frequently implemented on edge gateways (Akshya et al., 2025).

### 6.2 Hybrid CNN-LSTM intrusion detection models

The intelligent IoT intrusion detection using a hybrid CNN–LSTM architecture is shown in Figure 7. It uses Convolutional neural networks to extract spatial features and Long Short-Term Memory (LSTM) networks to capture the temporal relationships in the network traffic sequences. Input traffic undergoes convolutional layers, pooling of feature representations, sequential memory units, attention mechanisms and fully connected layers, and ultimately generates multi-class detector outputs to identify intrusions. This multi-layered structure allows the model to reflect local traffic properties as well as long-term behavior trends of complicated cyberattacks.

**Figure 7:**
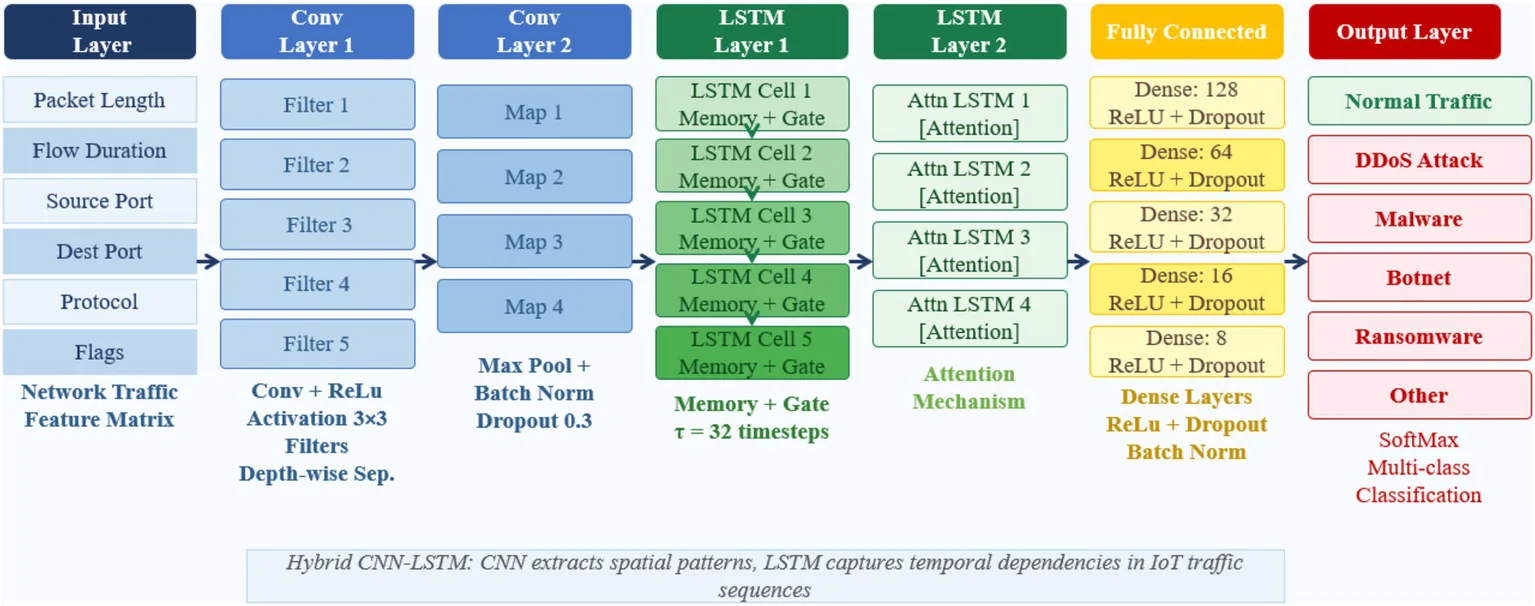
Hybrid CNN–LSTM architecture for IoT intrusion detection illustrating spatial feature extraction, temporal sequence modelling, attention mechanisms, fully connected classification layers, and multi-class attack prediction (created by the authors based on the reviewed literature).

Besides the model architecture, Figure 7 represents an intuition for hybrid deep learning approaches to outperform single-model architecture in the complex IoT environment. While convolutional layers are effective at extracting spatial structures within the identified network traffic features, LSTM layers help to retain temporal structures within the network traffic characteristics more effectively, which is key in capturing the temporal dynamics and behavior of various types of network attacks involving multiple stages, such as botnet communication, ransomware propagation, for example, and persistent malware. Attention mechanisms are also introduced to enhance feature prioritization, prioritizing the most informative temporal patterns when detecting intrusions. As a result, the hybrid CNN–LSTM framework is proven to offer a satisfactory balance between detection capability and sequential traffic analysis, which is the conceptual foundation for the hybrid deep learning models introduced in this section.

Hybrid neural networks are a combination of convolutional and recurrent networks that can take advantage of both. The success of such a combined approach is proved by a federated learning-based hybrid convolutional recurrent neural network to detect multiple classes of intrusion in IoT networks (Ali et al., 2024). This mixed method is especially efficient in identifying high-level cyberattacks associated with both structural abnormalities and the change in the temporal behavior (Von Der Assen et al., n.d.). HoleMal, a lightweight IoT malware scanning framework, that relies on efficient processing of host-level traffic, illustrates the benefits of temporal modelling of host-level traffic patterns on the accuracy of malware attribution (Hakiki et al., 2025). The cross-architecture Internet of Things malware detection and classification relies on the structural analysis capabilities of CNNs together with temporal modules (Hasan et al., 2022). An overview of beneficial neural networks reveals a new research problem in the area of deep learning security and IoT malware detection (Mansour et al., 2025). Resource and workload-aware malware detection using distributed computing in IoT networks also solves the deployment of heterogeneous groups of devices (Yama and Uda, 2025).

### 6.3 Generative models of anomaly detection

Generative models are a new trend in the research of IoT intrusion detection (Bonsu and Akekudaga, 2025). Explainable IoT security in real-time with machine learning and Conditional Tabular Generative Adversarial Network (CTGAN)-enhanced detection of resource-constrained devices illustrates how generative models can supplement small training sets (Wajahat et al., 2024). A visual encoding of binary features is used to create an optimized and efficient image-based IoT malware detection technique (Zhukabayeva et al., 2024). The generative models are especially effective in the detection of zero-day attacks since they do not need the labelled malicious samples to be trained. One example of an IoT edge-based IDS with combined models of ML and generative AI is given (Bonsu and Akekudaga, 2025).

### 6.4 Dataset-agnostic deep neural architectures

The alternative line of research is to come up with dataset-agnostic intrusion detection models. Image-based neural network classification to detect intrusion in IoT and wireless networks proves that often the better designed preprocessing pipelines, rather than more complex models, determine the detection performance (Ficco et al., 2024). A privacy-preserving BERT-based lightweight model of IoT and IIoT devices can be revolutionized by revolutionizing cyber threat detection with large language models (Mittal and Rajvanshi, n.d.).

## 7 Federated learning architectures for IoT intrusion detection

The overall federated learning process used for privacy-preserving IoT intrusion detection is shown in Figure 8. Distributed local model training is carried out in various IoT verticals such as smart homes, healthcare, industrial IoT, smart cities, and transportation systems, where the local collected data is used for training intrusion detection models without sharing raw data. Locally-trained parameters are securely communicated to a global aggregation server using privacy-preserving protocols such as Differential Privacy, Homomorphic Encryption, and Secure Multi-Party Computation to enable global model optimization while upholding data privacy.

**Figure 8:**
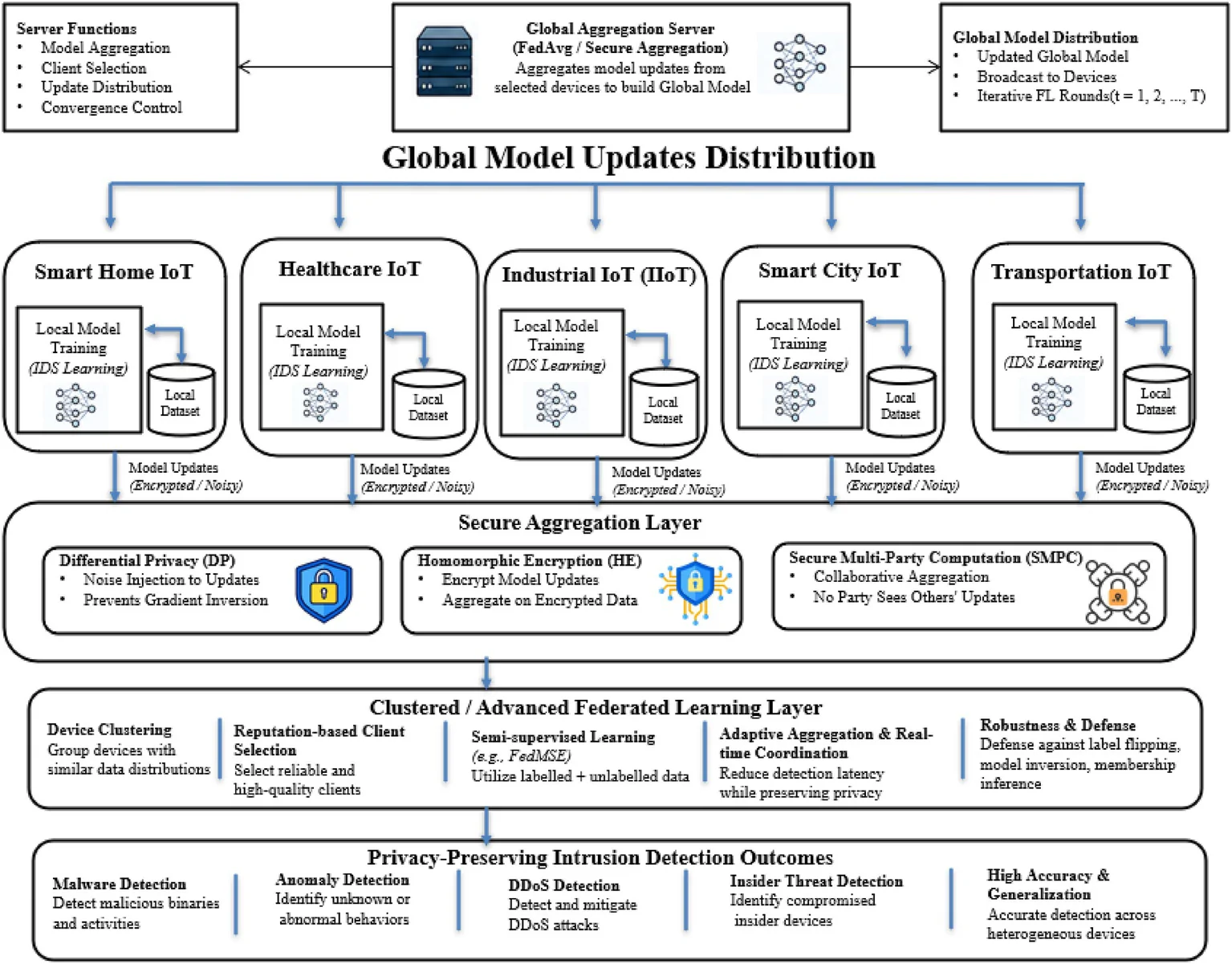
Federated learning workflow for privacy-preserving IoT intrusion detection illustrating distributed local model training, secure aggregation, advanced federated learning mechanisms, and collaborative intrusion detection across heterogeneous IoT environments (created by the authors based on the reviewed literature).

Aside from showing the workflow of federated learning, Figure 8 also shows how simple federated learning evolves to advanced collaborative architectures for intrusion detection. The figure illustrates the combined effects of federated learning, reputation-based client selection, semi-supervised learning, adaptive aggregation and robustness mechanisms in enhancing model convergence, scalability, robustness to adversarial attacks, and resilience in heterogeneous non-IID data distributions. Thus, the development of the analytical background for the secure aggregation techniques, privacy preserving mechanisms, and advanced federated learning strategies throughout this section are grounded in Figure 8.

The decentralized nature of the IoT ecosystem presently poses serious challenges to centralized security models (Mofidi et al., 2024). Although centralized security models are effective in enterprise environments, they become inefficient in IoT deployments because devices are geographically distributed, resource-constrained, and continuously generate sensitive data. Figure 8 illustrates the federated learning workflow that addresses these challenges through distributed collaborative model training.

Figure 1 provides the overall system architecture, whereas Figure 8. focuses specifically on the federated learning workflow. In the architecture shown in Figure 1, the Local Federated Aggregator located at the Edge/Fog layer coordinates collaborative model training by collecting local model updates from distributed devices instead of raw data. This design enhances privacy while introducing new security challenges such as model poisoning and gradient leakage attacks.

Figure 4 identifies model poisoning and gradient leakage as representative data-oriented attacks that directly threaten federated learning systems, motivating the secure aggregation, privacy-preserving learning, and adversarial defense mechanisms discussed in this section.

The transmission of raw information from a device to centralized servers in most IoT applications presents communication overhead, as well as privacy risks (Bekkouche et al., 2022). Federated learning is a prospective paradigm that allows devices to learn using machine learning models on-device and only exchange model parameters but not data (Khan et al., 2024). Federated learning minimizes communication overhead by decentralizing the training process, maintaining privacy and scaling to large IoT deployments (Jamshidi et al., 2025). A thorough survey establishes that federated learning in intrusion detection in the IoT environment offers a solid privacy-preserving approach (Almobaideen et al., 2025).

### 7.1 Fundamentals of federated learning

Federated learning functions based on an iterative training process where each device has a local dataset and trains its models independently. The aggregation server and the connection between the aggregation server and the participating devices combine the received updates with the help of algorithms like Federated Averaging and subsequently redistribute the global model to the participating devices (Khan et al., 2024). A two-stage hybrid federated learning architecture of privacy-preserving IoT anomaly detection illustrates the way that iterative aggregation can be designed to support the case of multi-class detection (Chen Z. et al., 2025). The Federated Learning for Anomaly Detection in IoT Networks (FLADEN) model of federated learning-based anomaly detection of IoT networks shows how the regular FL workflows can be utilized in unsupervised anomaly detection environments (Portales and Riegler, 2024). Federated learning based IoT malware analysis gives a thorough overview of federated paradigm-based malware analysis (Kasarapu et al., 2024).

### 7.2 Federated learning for privacy-preserving IoT security

One of the driving forces behind the use of federated learning in the security of IoT is privacy preservation. Monitoring systems in healthcare, industrial automation systems and smart city systems produce sensitive data about operations (Alanezi et al., 2026). Deployed in the context of urban-scale FL, the demonstration of advanced artificial intelligence and federated learning structures of privacy-preserving cyberthreat detection in IoT-assisted sustainable smart cities shows how FL can be implemented at a large scale (Hasan and Tasnim, 2025). As devices do not transmit raw data, but only model parameters, the threat of sensitive data being revealed is considerably minimized. The privacy principles can be extended to device-level communication with lightweight and privacy-conscious device-to-device authentication of smart healthcare systems based on the IoT (El-Ghamry et al., 2023). The trade-offs between privacy mechanisms, communication cost and robustness across different federated learning variants are summarized in Table 9.

**Table 9:** Federated learning variants comparison for IoT intrusion detection.

FL variant	Aggregation method	Privacy mechanism	Communication cost	Adversarial robustness	Non-IID handling	Refs
Standard FL	FedAvg	None/Basic	Moderate	Low	Poor	Khan et al. (2024)
FL + Differential Privacy	Noisy FedAvg	Differential Privacy	Moderate	Moderate	Moderate	Hendaoui et al. (2025); Venkatasubramanian et al. (2023)
FL + Homomorphic Encryption	Encrypted Aggregation	Homomorphic Encryption	High	High	Moderate	Venkatasubramanian et al. (2023)
FL + Secure Multi-Party Computation	SMPC Aggregation	Secure Computation	High	High	Moderate	Mahmood et al. (2025)
Clustered FL	Per-Cluster FedAvg	Basic	Moderate	Moderate	High	Ragab et al. (2025)
Semi-Supervised FL (FedMSE)	MSE-Weighted Aggregation	Basic	Low	Moderate	High	Das et al. (2024)
Two-Stage Hybrid FL	Stage-wise Aggregation	Layered Privacy	Moderate	High	High	Shahin et al. (2025); Rampone et al. (2025)
Reputation-Based FL	Weighted Aggregation	Trust Scoring	Low	High	High	Bensaid et al. (2025)
Mini-Batch FL	Mini-Batch FedAvg	Basic	Very Low	Low	Moderate	Alrayes et al. (2025)
Blockchain-Integrated FL	Ledger-Verified Aggregation	Blockchain Consensus	High	Very High	Moderate	Yan and Khoei (2025); Smmarwar et al. (2023)

Industrial IoT an example is that in fog-assisted IoT smart homes, securing the environment using a federated learning-based intrusion detection framework proves that FL is effective in ensuring data confidentiality among the devices deployed by the fog (Ferrag et al., 2024). Nevertheless, even with these benefits, model updates sent throughout training can still indicate biased information about local datasets. This problem is resolved by a privacy-preserving IoT network based on statistical learning and optimization algorithm (Sharma and Yadav, 2025). The comparative analysis of methods and applications of privacy-preserving security of IoT networks offers a general assessment of privacy assurances with varied FL configurations (Shahin et al., 2025). A resource-nesting, privacy-preserving federated edge learning system is yet another example of how Federated learning can be implemented in real-time Internet of Medical Things applications, providing further evidence to the practicality of FL in time-sensitive medical applications (Alanezi et al., 2026).

### 7.3 Secure aggregation mechanisms

To ensure the safety of model updates being exchanged during federated training, secure aggregation mechanisms are created. Malware detection using AI and differential privacy to achieve zero trust security in Internet of Things networks proves that with noise injection meticulously adjusted, individual data samples cannot be extracted by aggregating gradients (Hendaoui et al., 2025). A homomorphic encryption-based privacy-preserving federated learning-based intrusion detection method of cyber-physical systems is used to enable the aggregation of encrypted model updates (Venkatasubramanian et al., 2023). Secure multi-party computation is integrated with visual feature encoding in a federated intrusion detection system that uses visual encoding to encode and lightweight secure aggregation to reduce the cost of communication and privacy loss in the aggregation process of the IoT (Mahmood et al., 2025).

### 7.4 Clustered and advanced federated learning architectures

The non-identically and non-independent nature of the IoT data is one of the biggest challenges in federated learning systems. The clustered federated learning architecture of network anomaly detectors in large heterogeneous IoT networks has been adopted to solve this problem by clustering together similar devices with regard to data distributions (Ragab et al., 2025).

FedMSE, a semi-supervised federated learning-based IoT network intrusion detection system, builds on the clustered FL paradigm by generalizing to cases where only a part of the training data has labels (Nguyen and Beuran, 2025). Strengthening federated learning for anomaly detection in IoT through reputation-based client selection shows that the selective inclusion of quality participating devices enhances global model convergence (Alfadhil et al., 2025). A hybrid federated learning-based framework for privacy preserving near-real-time intrusion detection in IoT shows an example of adaptive aggregation to minimize detection latency without compromising privacy (Rampone et al., 2025). Federated learning-based robust android malware detection label flipping attacks and defenses deal with adversarial manipulation of model changes (Eslamnejad et al., 2025). The improvement of privacy-saving intrusion detection using federated learning adds other mechanisms to solve model inversion and membership inference attacks (Alazab et al., 2023).

Improved IoT security, based on a privacy-sensitive federated learning model to detect intrusions in real-time with accuracy across devices, shows performance improvements (Puviarasu and Sudha, 2026). Federated learning is a privacy-preserving method of distributed intrusion detection, secured by Internet of things devices (Al Amro, 2025). The problem of insider threat detection is specifically tackled with privacy-preserving federated learning to gain the advantage in identifying compromised insider devices in IoT networks (Kamatchi and Uma, 2025).

FL-IDS, a federated learning-based intrusion detection system over edge transport IoT, shows that FL is applicable to the transportation infrastructure of safety-critical applications (Bhavsar et al., 2024). A federated learning-based malware detection model with SIM-FED, a secure IoT malware detector, further incorporates specific malware detection capabilities (Nobakht et al., 2024). Lightweight intrusion detection systems federated with learning applied to the wireless sensor network offer solutions to cybersecurity in smart city settings in terms of DDoS attacks (Devi et al., 2025). Privacy-sensitive federated learning-based intrusion detection scans the entire landscape of FL variants used in this area (Bhatia et al., n.d.).

## 8 TinyML and edge intelligence for IoT security

TinyML is a new discipline that aims to implement machine learning models on computers approximately the size of a microcontroller. TinyML can run complicated neural network models on tight hardware constraints by compressing them via quantization, pruning and sharing of parameters (Ramadan et al., 2025). The federated learning of IoT devices that improves TinyML with on-board training proves the point that devices can also improve their own models without depending on the cloud (Ficco et al., 2024). TinyML can be applied in IoT security applications to perform intrusion detection at the device level, whereby devices examine their own behavior in real-time. Federated learning and TinyML on IoT edge devices review the challenges, progress and future of this booming arena (Ramadan et al., 2025).

### 8.1 On-device intrusion detection

On-device detection allows devices to track network traffic patterns, system calls and behavioral patterns without the assistance of external infrastructure (Ficco et al., 2024). Devices can invoke local mitigation measures or send notifications to upper-level security systems when an abnormal behavior is detected (Ramadan et al., 2025).

### 8.2 Federated TinyML systems

Recent research combines federated learning with TinyML technologies to create decentralized collaborative security frameworks (Ficco et al., 2024). The model updates are then sent to aggregation servers where global models are built (Ramadan et al., 2025).

### 8.3 Energy-efficient learning strategies

IoT security systems heavily depend on energy efficiency since a significant portion of devices use batteries. A system of attack detection through an energy-based survey of IoT devices classifies and weighs the use of detection techniques based on their energy consumption (Merlino and Allegra, 2024). The analysis of machine learning-based intrusion detection systems in IoT in terms of performance and energy usage proves the extreme significance of collectively optimizing detection accuracy and energy overhead (Jamshidi et al., 2025).

Mini-batch federated learning reduces computational complexity by training models on smaller subsets of data. A lightweight mini-batch federated learning approach for attack detection in the IoT demonstrates that even small data batches produce models competitive with full-batch training (Ahmad and Shah, 2024). Client selection mechanisms improve efficiency by selecting only a subset of devices to participate in each training round. Enhancing federated learning through reputation-based client selection prioritizes devices with sufficient resources and stable connectivity (Alfadhil et al., 2025).

## 9 Edge–Fog–Cloud collaborative security architectures

The current IoT security architectures are based more on hierarchical computing architectures that spread intelligence at various levels. The stratification of intelligence in ML-based anomaly detection by incorporating IoT, cloud and edge computing illustrates that intelligence may be scaled to the abilities of each layer. These architectures generally comprise three main layers (Rey et al., 2022). IoT device layer, edge or fog computing layer and cloud intelligence layer (Wakili and Bakkali, 2025).

The complete hierarchical architecture is illustrated in Figure 1. The figure demonstrates how security intelligence is distributed across the IoT Device Layer, Edge/Fog Computing Layer, Network Infrastructure, and Cloud Intelligence Layer. This layered organization enables computational tasks to be executed at the most appropriate location, balancing latency, scalability, and privacy requirements.

### 9.1 Device-level security monitoring

The bottom layer of the architecture is the IoT device layer. Simple security monitoring is done by devices with lightweight anomaly detection algorithms or TinyML models (Nawshin et al., 2024). To show device-level monitoring at scale, the problem of enhancing IoT resilience at the edge with a resource-efficient anomaly detection framework in real-time on streaming data is addressed (Mahmud et al., 2024). These models interpret local measurements of sensors, network traffic patterns and system behavior to detect suspicious activities. Since device-level detection is performed at the very point of data creation, it can give real-time feedback on outliers (Mabrouk et al., 2025).

### 9.2 Edge intelligence to real-time analysis

The edge computing layer is a processing layer that lies between the cloud infrastructure and IoT devices. The adaptive federated edge intelligence to detect cyberthreats in real-time in resource-limited IoT settings proves that lightweight deep learning models can be successfully deployed to the edge (Wakili and Bakkali, 2025). Edge nodes combine information from several devices and apply deep learning models that can analyze the intricate network traffic patterns (Rey et al., 2022). An example of edge-based detection based on programmable network management with software-defined IoT network intrusion detection and mitigation framework is presented using AI (S’aez-de-C’amara et al., 2023). The importance of the fog layer as an intermediary to offload computation while maintaining data confidentiality is shown through the development of encryption-based fog computing to enhance privacy and security in IoT-based smart grid systems (Nguyen and Beuran, 2025).

### 9.3 Cloud-based threat intelligence

The cloud offers security intelligence in the world. An example of how cloud-hosted virtual functions may coordinate the detection of malware in large IoT-based networks is a network function virtualization system to detect malware in large networks (Alfadhil et al., 2025). Security systems on the cloud use aggregated information, obtained across multiple edge nodes, to detect patterns of attack around the globe. Federated learning artificial intelligence at the advanced stage of smart city IoT environments is based on cloud analytics to correlate threat intelligence in city-scale deployments (Hasan and Tasnim, 2025).

IoT-based smart grid security and privacy-preserving frameworks and applications rely on cloud infrastructure to sustain global threat models alongside adherence to data protection requirements (Rampone et al., 2025). Statistically analyzing machine-type communication data sets allows a more precise baseline profile at the cloud level through modelling of IoT traffic patterns (Puviarasu and Sudha, 2026).

## 10 Blockchain-based security frameworks for IoT intrusion detection

Figure 9 presents the blockchain-integrated federated learning trust framework considered throughout this review for secure IoT intrusion detection. The architecture combines edge intelligence, federated learning, blockchain governance, and explainable AI into a unified security framework. Local intrusion detection models are first trained at distributed edge nodes before validated model updates are securely aggregated through federated learning. Blockchain smart contracts subsequently verify device identities, validate model updates, maintain reputation scores, enforce access control policies, and record threat intelligence using an immutable distributed ledger. This global security intelligence empowers explainable AI analytics and coordinated security decision making in a diverse IoT and Industrial IoT landscape.

**Figure 9:**
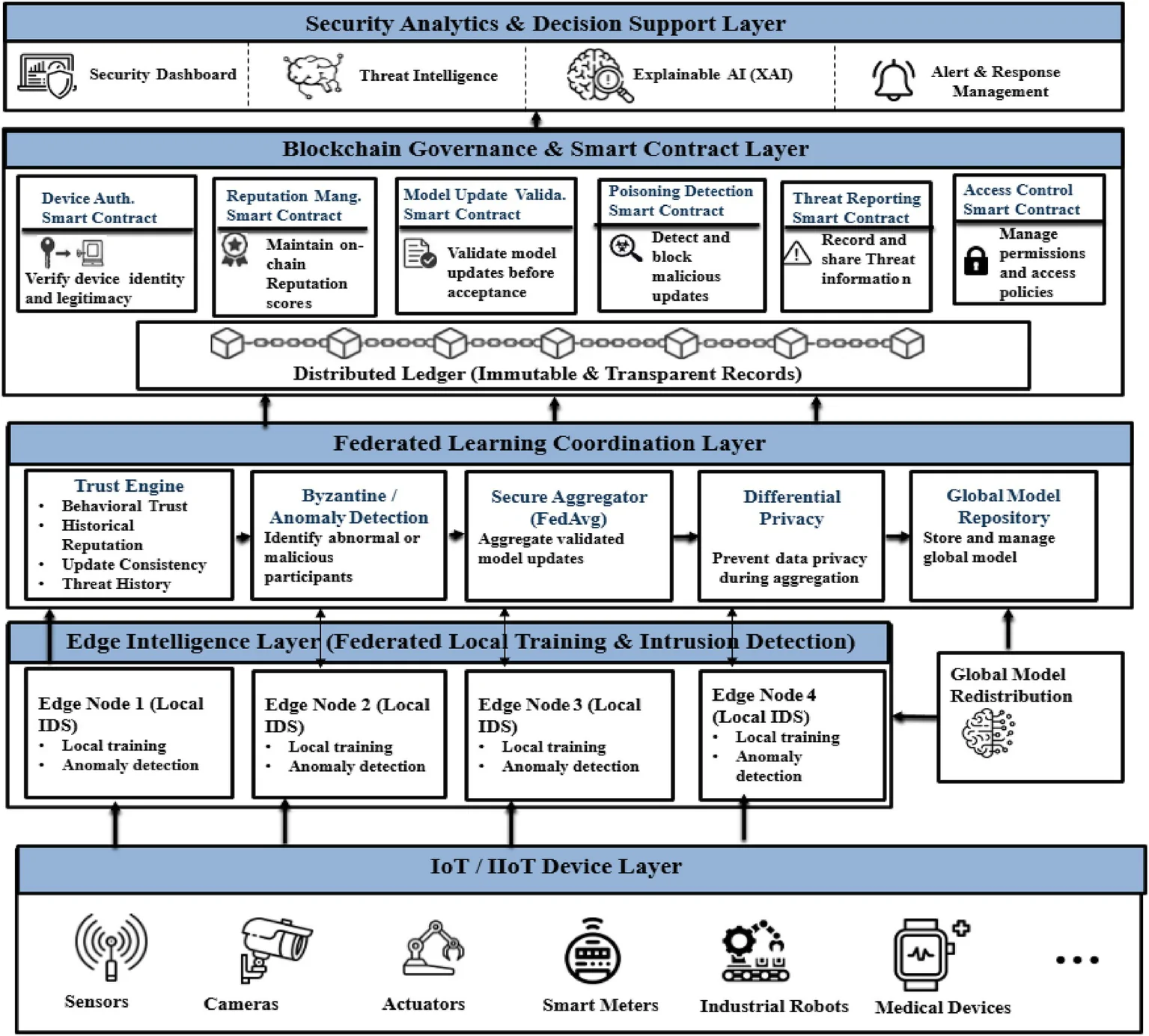
Blockchain-integrated federated learning trust framework for IoT intrusion detection illustrating edge intelligence, federated learning coordination, blockchain-enabled trust management, smart contract governance, and explainable security analytics (created by the authors based on the reviewed literature).

Figure 9 also shows the usage of blockchain in collaborative intrusion detection, where blockchain is used to add decentralized trust management to federated learning, to illustrate the architectural workflow. It not only provides secure aggregation of models but also leverages smart contracts, client reputation for improved selection, client poisoning detection, and immutable audit trails to ensure the integrity, transparency, and accountability of distributed learning. The figure also highlights the need for the machine learning (ML) model to be accurate, trustworthy and to provide secure model validation and explainable security analytics for successful IoT intrusion detection. Guided by this, in this section the concept of trust mechanisms based on blockchain is introduced throughout the figures.

The growing complexity of IoT ecosystems has underscored the weaknesses of centralized security infrastructures. Edge-based intrusion detection methods using blockchain and federated learning in industrial IoT networks offer a review of how blockchain can overcome centralized trust issues (Ali et al., 2024). Blockchain offers a distributed ledger architecture where transactions are stored in immutable blocks verified by consensus mechanisms, enabling secure and decentralized IoT intrusion detection workflows as illustrated in Figure 9. The use of deep neural networks and blockchain-based cybersecurity to improve anomaly detection and prevention in IoT proves this architecture in practice (Sathyabama and Katiravan, 2025). In the framework of IoT security, blockchain allows decentralized authentication of security data between multiple nodes (Ali et al., 2024).

As illustrated in Figure 1, Blockchain Trust Management is positioned in the Cloud Intelligence Layer, creating decentralized trust between edge nodes that are distributed and participants in federated learning. Whereas the federated architecture is complemented by blockchain as it ensures integrity of model updates and stronger trust management through the IoT ecosystem.

Referring to Figure 4, blockchain primarily addresses data integrity, model authenticity, and trust management challenges by protecting collaborative model updates, preventing unauthorized modifications, and supporting secure authentication among distributed IoT nodes.

### 10.1 Blockchain-integrated federated intrusion detection

Integration with federated learning systems is one of the most important blockchain-based applications in IoT security. The blockchain provides a safe system to authenticate model updates, where each update is logged as a transaction in the ledger and authenticated before being included in the global model (Ali et al., 2024). This architecture prevents malicious participants from injecting contaminated model parameters into the aggregation process (Eslamnejad et al., 2025).

### 10.2 Smart contracts for security automation

Smart contracts are automated scripts that run when specific conditions are met. Smart contracts have the potential to automate the validation of model updates, threat reporting and authentication of devices in IoT security architectures (Sathyabama and Katiravan, 2025). Systems of device reputation management can also be automated by use of smart contracts. Lightweight, trust and privacy collaborative IoT intrusion detection shows the potential to use reputation scores calculated on-chain to control the involvement of devices in collaborative detection (Wardana et al., 2024).

### 10.3 Benefits and limitations of Blockchain-based IoT security

Blockchain implementation improves transparency through recording all security-related transactions in an unchangeable registry, removes single points of vulnerability and offers audit trails that are difficult to alter. Nonetheless, consensus mechanisms have the potential to add computational and network latency, which can reduce scalability in very large IoT systems. To minimize this overhead, lightweight blockchain frameworks and alternative consensus algorithms have been suggested (Ali et al., 2024).

## 11 Trust-aware intrusion detection frameworks

When it comes to the large-scale IoT setup, not every device involved can be expected to be reliable. Trust Enhanced Anomaly Detection (TEAD), a trust-enhanced intrusion detection framework for IoT-enabled wireless sensor networks, copes with it by considering trust assessment in the detection chain (Al Amro, 2025). Lightweight, trust-managing and privacy-preserving collaborative intrusion detection of the Internet of Things further generalizes trust management to collaborative detection scenarios (Kamatchi and Uma, 2025). Trust-aware systems provide dynamic trust scores to devices according to their past behavior, communication patterns and data integrity. The trust score can be used to control access to data at the edge, as evidenced by a federated edge intelligence framework with trust-based access control of secure and privacy-preserving IoT systems (Bhavsar et al., 2024).

The Trust Engine shown in Figure 9 provides the basis for the trust-aware intrusion detection mechanisms discussed in this section by maintaining behavioral trust, reputation scores, and historical security information for participating devices.

### 11.1 Trust score computation

Trust scores are usually calculated by various behavioral measures such as frequency of communication, success rate of packet delivery, consistency of data and energy usage patterns (Al Amro, 2025). The development of IoT security based on an elaborate AI-driven trust system to detect intrusions shows the potential of machine learning to assess the multidimensional trust metrics on a dynamic basis (Nobakht et al., 2024). Trust computation models are a statistical analysis-based machine learning framework that determines behavioral deviations. The system can adjust to the dynamic nature of a network by maintaining trust scores through repeated updates with observed behavior (Bhavsar et al., 2024).

### 11.2 Integration of trust with intrusion detection

Trust-conscious intrusion detection systems incorporate trust scores as part of anomaly detection techniques. Low-trust devices can either be monitored further or can be left out of collaborative learning (Al Amro, 2025). In the process of federated learning, the aggregation server can give lower weights to model updates posted by devices that have low trust scores. This weighted aggregation strategy is applied by enhancing federated learning to detect anomalies in IoT-based systems by reputation-based client selection (Bensaid et al., 2025). The integration of trust evaluation and anomaly detection creates a multi-layer security architecture that improves resilience against insider attacks and compromised devices (Nobakht et al., 2024). A signature and anomaly-based intrusion detection system for secure IoTs and V2G communication illustrates how hybrid detection strategies incorporating trust awareness perform across heterogeneous IoT deployments (Reji et al., 2025).

## 12 Explainable artificial intelligence in IoT security

Although artificial intelligence models have greatly enhanced intrusion detection abilities, some deep learning models are black-box systems. Explainable AI-based intrusion detection for securing IoT-enabled autonomous transportation systems illustrates how XAI can help to close the gap between model performance and operational transparency (Akshya et al., 2025). Explainable AI overcomes this issue by giving details on how machine learning models make decisions. Federated Learning of Explainable AI (FedXAI) for deep learning-based intrusion detection in IoT networks further applies explainability to the federated learning framework (Kalakoti et al., 2025).

Figure 1 also highlights the XAI Security Analytics component within the Cloud Intelligence Layer. Explainable AI improves the transparency and interpretability of AI-driven intrusion detection decisions, allowing security analysts to better understand model predictions and increasing confidence in automated threat detection systems.

Figure 2 further illustrates Explainable AI as a cross-cutting security mechanism that improves the interpretability of AI-driven intrusion detection across network-level, device-level, and data-oriented attack scenarios, enabling security analysts to better understand automated detection decisions.

Figure 9 further illustrates that Explainable AI operates within the Security Analytics and Decision Support Layer, where interpretable model predictions complement blockchain-verified trust information to support transparent security decisions.

### 12.1 Global model interpretability using SHAP

Shapley Additive Explanations (SHAP) gives weights to each feature based on the amount it adds to the model predictions. SHAP analysis in intrusion detection systems determines what attributes of network traffic are most influential towards detecting an attack (Akshya et al., 2025). Transfer learning with XAI for robust malware and IoT network security illustrates how SHAP visualizations can reveal the discriminative features learned in transfer learning (Almadhor et al., 2025).

### 12.2 Local interpretability using LIME

Local Interpretable Model-Agnostic Explanations (LIME) is a method that gives explanations of individual predictions by locally approximating the model with a more interpretable model. Explainable Machine Learning-based Android Malware Detection (EML-AMD), an explainable machine learning framework for detecting adaptive Android malware, shows that local interpretability methods lead to better analyst trust in automated detection decisions (Ahmad and Shah, 2024; Patel and Ghosh, 2025). SHAP world analysis with LIME local explanations gives a complete interpretability. This dual-layer approach improves transparency and facilitates regulatory compliance in the case where automated decision-making should be audited (Eslamnejad et al., 2025).

## 13 Dataset engineering for IoT intrusion detection

Availability of high-quality and diverse datasets is critical to the effectiveness of machine learning-based intrusion detection systems, with key IoT security datasets and their characteristics summarized in Table 10. Recent data on network flooding attacks within Software-Defined Networking (SDN)-based IoT systems shows how dataset-specific creation can further the development of specialized detection systems (Karmous et al., 2025). A large-scale digital forensics dataset known as Cyberattack Attribution Detection (IoT-CAD), which provides AI-based techniques of cyberattack attribution detection in IoT settings, resolves the issue of attributing attacks to particular threat actors (Mohamed et al., 2025). Current studies have paid attention to the construction of realistic datasets based on IoT testbeds that replicate real interactions between devices and cyberattack situations. A way to solve the problem of smart device profiling is by creating a large-scale IoT Z-Wave intrusion detection dataset (Shafi and Lashkari, 2025).

**Table 10:** Summary of IoT security datasets.

Dataset/source	Year	Protocols covered	Attack types	Data modality	Key feature	Refs.
ToN-IoT	2024	MQTT, HTTP, DNS	DDoS, Injection, Ransomware, Scan	Network + OS Logs + Telemetry	Industry 4.0 testbed, multi-layer	Merlino and Allegra (2024)
MQTTEEB-D	2025	MQTT	Flooding, Auth Attack, Malformed Packet	Network Traffic	Real-world MQTT-specific dataset	Rahmati and Rahmati (2025)
IoT-Z-wave (IDS Dataset)	2025	Z-Wave	Intrusion, Profiling Attacks	Device Traffic + Behavior	Smart device profiling, Z-Wave specific	Kirubavathi et al. (2025)
IoT-CAD	2024	Multi-protocol	Attribution-Labelled Cyberattacks	Network + Forensic Logs	Cyberattack attribution focus	Alotaibi (2025)
SDN-IoT Flooding Dataset	2025	SDN/OpenFlow	Network Flooding	SDN Controller Logs	SDN-based IoT environment	Shruti et al. (2024)
MTC Statistical Dataset	2025	MTC/LTE	Traffic Anomalies	Statistical Traffic Features	IoT traffic pattern modelling	Puviarasu and Sudha (2026)

### 13.1 Multi-protocol IoT datasets

IoT networks have many different communication protocols, such as Message Queue Telemetry Transport (MQTT), Hypertext Transfer Protocol (HTTP), Constrained Application Protocol (CoAP), ZigBee, Zensys Wave (Z-Wave) and Modbus. Detailed security datasets should be able to reflect actual environments by capturing traffic of various protocols (Aqachtoul et al., 2025). Statistical modelling of IoT traffic patterns based on statistical analysis of Machine-Type Communication (MTC) datasets gives a critical understanding of multi-device IoT traffic characteristics (Ruiz-Guirola et al., 2025). Multi-protocol datasets combine network traffic logs, device telemetry, system calls and application-level data. These layers combine to create more robust intrusion detection models (Mohamed et al., 2025).

### 13.2 MQTT-IoT security datasets

MQTT is a simple messaging system common in IoT systems. A real-world IoT cybersecurity dataset, MQTTEEB D, used in AI-driven threat detection in MQTT networks, contains attack scenarios including message flooding, authentication attacks, malformed packet injection and denial-of-service attacks (Aqachtoul et al., 2025).

### 13.3 The ToN-IoT dataset architecture

The ToN-IoT dataset is one of the most detailed datasets for IoT intrusion detection research and simulates a full Industry 4.0 environment. The ToN-IoT dataset can be used to create a new intrusion detection system to cover industrial IoT devices, proving that it is a multi-dimensional benchmark. The dataset contains several attack scenarios, including distributed denial-of-service attacks, data injection attacks, scanning activities and ransomware behavior, which allows multi-dimensional intrusion detection research (Cao et al., 2025).

## 14 Comparative evaluation and critical analysis of IoT intrusion detection approaches

The creation of intrusion detection systems for IoT ecosystems has generated a broad spectrum of security architectures, which differ in learning strategies, deployment models, privacy guarantees and computational requirements. An overview of network intrusion detection across both IoT and non-IoT paradigms offers a comparative baseline (Abdulkareem et al., 2024). Conventional machine learning-based intrusion detection systems are based on centralized training environments. Though such models have high detection rates, their centralized structure incurs communication overhead and privacy issues (Ozdogan, 2024). Large-scale IoT deployments suffer serious latency due to the need to pass raw device data to centralized servers (Vyas et al., 2024).

Deep learning detection frameworks enhance detection, as they are able to capture nonlinear relationships in network traffic patterns. Convolutional neural networks and recurrent neural networks show good performance in identifying botnet communication patterns and sequential attack patterns (Chaganti et al., 2022). Nevertheless, such models can have high computational requirements (Jamshidi et al., 2025). Lightweight machine learning models overcome this drawback through simplifying the models and streamlining feature selection procedures (Esmaeilyfard et al., 2025). IoT-based DDoS attack detection methods using AI offer taxonomies and extensive surveys of lightweight paradigms in a variety of attack types (Bala and Behal, 2024). The development from classical ML to new quantum-enhanced detection paradigms is described in a systematic review of anomaly detection in IoT security toward quantum machine learning (Aparcana-Tasayco et al., 2025).

Federated learning is used in intrusion detection systems (IDSs) to enable multiple devices to collaboratively train models without sharing raw data, thus ensuring privacy protection (Khan et al., 2024). However, federated systems suffer from overhead communication and stability problems in the convergence of the models and the susceptibility to adversarial manipulation (Ragab et al., 2025). Blockchain-based intrusion detection systems (IDSs) bolster security measures by establishing a decentralized trust framework and ensuring audit trails are tamper-proof (Yan and Khoei, 2025). Multiple defense layers (Ramadan et al., 2025) are covered in the enhancement of network security in IIoT by a deep dive into AI enabled intrusion detection systems. These have been combined to create hybrid architectures. For the IoT ecosystem, AI-based intrusion detection can provide a detailed analysis of the combined paradigm’s capabilities of achieving superior results over single paradigms (Kumar et al., 2022). The future of IoT security is said to involve more of a collaboration between machine learning and quantum computing that can convey ideas, attacks, and issues (Kumar and Chittora, 2024).

### 14.1 Comparison of experimental datasets

Most studies reviewed in this survey evaluated intrusion detection performance using publicly available benchmark datasets such as ToN-IoT, CICIDS2017, BoT-IoT, CICIoT2023, and UNSW-NB15 (Sharma and Bairwa, 2025; Cao et al., 2025). Although these datasets facilitate reproducible benchmarking, they are predominantly collected under laboratory conditions and therefore may not fully represent heterogeneous Industrial IoT environments (Shahin et al., 2024; Aparcana-Tasayco et al., 2025). Furthermore, only a limited number of studies validated their models using real deployment data collected from operational edge devices or industrial infrastructures (Ramadan et al., 2025; Villafranca and Cano, 2025). Consequently, future research should emphasize standardized benchmarking using realistic multi-domain datasets that better reflect practical deployment scenarios (Sharma and Bairwa, 2025; Aparcana-Tasayco et al., 2025).

### 14.2 Comparison of evaluation metrics

Detection accuracy is the most commonly reported metric for all types of intrusion detection systems, be they ML, DL, FL, TinyML, and blockchain-based. However, complementary measures, such as Precision, Recall, F1-score, Matthews Correlation Coefficient, False Alarm Rate, inference latency, memory utilization and energy consumption are not reported in many studies. The data sets generated by IoT systems are usually highly imbalanced and using accuracy alone may overestimate the effectiveness of the model. A consistent multi-metric assessment protocol should therefore be used in future evaluations. Table 11 summarizes the reported statistical validation techniques for all 114 of the reviewed studies to further evaluate the rigor of the experimental evaluation across the literature.

**Table 11:** Summary of statistical validation across the 114 reviewed studies.

Statistical validation	Papers (*n* = 114)	Percentage
Accuracy reported	111	97.4%
Accuracy + Std. Dev.	44	38.6%
Accuracy + Confidence Interval	8	7.0%
Accuracy + Significance Test	22	19.3%
Accuracy + Cross-Validation	35	30.7%
Accuracy + At least one Statistical Validation	53	46.5%

To provide paper-wise evidence supporting the overall statistical analysis presented in Tables 11, 12, details the statistical validation methods reported by individual studies, including standard deviation, confidence intervals, significance tests, and cross-validation strategies.

**Table 12:** Statistical validation reported in the reviewed studies.

References	Accuracy	Std. dev.	Confidence interval	Significance test	Cross-validation
Guizani and Ghafoor (2020)	99.97% (best reported)	–	–	–	K-fold
Smmarwar et al. (2023)	93.2% (best reported)	±0.4%	–	–	–
Ngo et al. (2020)	Not explicitly stated	–	–	–	5-fold
Nazir et al. (2023)	100.0% (best reported)	–	–	–	10-fold
Lefoane et al. (2025)	99.1% (best reported)	–	–	–	–
Ozdogan (2024)	Not explicitly stated	–	–	–	–
Sharma and Bairwa (2025)	99.98% (best reported)	–	–	Wilcoxon signed-rank test, *p* < 0.01	Train-test split only
Chaganti et al. (2022)	98.5% (best reported)	–	–	–	–
Abualghanam et al. (2025)	99.14% (best reported)	–	–	–	–
Rey et al. (2022)	Not explicitly stated	–	[3, 4%] (95% CI)	Chi-square test	Train-test split only
Alazab et al. (2023)	99.92% (best reported)	–	[36, 37%] (95% CI)	–	Train-test split only
Ramadan et al. (2025)	100.0% (best reported)	–	–	ANOVA	Train-test split only
Von Der Assen et al. (n.d.)	96.3% (best reported)	–	[0, 1%] (95% CI)	–	Train-test split only
Devi et al. (2025)	100.0% (best reported)	±1.0%	–	Chi-square test	5-fold CV
Alhamdi et al. (2025)	100.0% (best reported)	–	–	ANOVA	Train-test split only
Khan et al. (2024)	86.12% (best reported)	–	[94, 95%] (95% CI)	–	–
Mofidi et al. (2024)	98.2% (best reported)	±0.6%	-	Paired *t*-test	–
Yan and Khoei (2025)	99.97% (best reported)	–	–	Paired *t*-test, *p* < 0.01	–
Singh and Siddalingaiah (2025)	99.05% (best reported)	–	[38, 39%] (95% CI)	–	–
Ciaramella et al. (2026)	100.0% (best reported)	–	–	–	5-fold CV
Khraisat et al. (2025)	99.0% (best reported)	±0.5%	–	–	–
Villafranca and Cano (2025)	94.5% (best reported)	±1.0%	–	–	Train-test split only
Kalakoti et al. (2025)	95.5% (best reported)	±10.0%	–	–	Train-test split only
Mehmmod et al. (2025)	99.93% (best reported)	–	–	*p*-value reported (*p* < 0.05)	Train-test split only
Chen J. et al. (2025)	99.57% (best reported)	–	–	ANOVA	Train-test split only
Hakiki et al. (2025)	94.2% (best reported)	–	–	–	–
Mansour et al. (2025)	99.54% (best reported)	–	–	ANOVA	K-fold
Bonsu and Akekudaga (2025)	95.05% (best reported)	–	[2, 3%] (95% CI)	–	Train-test split only
Jamshidi et al. (2025)	98.11% (best reported)	–	–	–	–
Chen Z. et al. (2025)	Not explicitly stated	–	–	ANOVA	Train-test split only
Sharma and Yadav (2025)	Not explicitly stated	–	–	Paired *t*-test	Train-test split only
Shahin et al. (2025)	99.0% (best reported)	–	–	–	–
Mahmood et al. (2025)	99.96% (best reported)	–	[1, 3%] (95% CI)	–	Train-test split only
Alrayes et al. (2025)	99.88% (best reported)	–	–	–	–
Nawshin et al. (2024)	100.0% (best reported)	–	–	–	10-fold
Mahmud et al. (2024)	95.31% (best reported)	–	–	Chi-square test	Train-test split only
S’aez-de-C’amara et al. (2023)	99.88% (best reported)	–	–	Chi-square test	Train-test split only
Nguyen and Beuran (2025)	Not explicitly stated	–	–	Chi-square test	Train-test split only
Alfadhil et al. (2025)	Not explicitly stated	–	–	Chi-square test	Train-test split only
Rampone et al. (2025)	99.98% (best reported)	–	–	–	–
Puviarasu and Sudha (2026)	99.97% (best reported)	–	–	–	K-fold CV (fold count N/A)
Al Amro (2025)	Not explicitly stated	–	–	ANOVA	–
Kamatchi and Uma (2025)	Not explicitly stated	–	[0, 1%] (95% CI)	*p*-value reported (*p* = 0.5)	Train-test split only
Nobakht et al. (2024)	99.61% (best reported)	–	–	–	–
Kumar and Chittora (2024)	99.99% (best reported)	–	[1, 2%] (95% CI)	–	5-fold
Anwer et al. (2025)	Not explicitly stated	±0.0033%	–	–	10-fold
Padmavathi and Saminathan (2025)	99.91% (best reported)	–	[3, 4%] (95% CI)	–	K-fold
Kaliappan et al. (2024)	93.0% (best reported)	–	[4, 7%] (95% CI)	–	Train-test split only
Almadhor et al. (2025)	98.85% (best reported)	±0.49%	–	Independent samples *t*-test	Train-test split only
Karmous et al. (2025)	Not explicitly stated	–	[34, 35%] (95% CI)	Paired *t*-test	–
Shafi and Lashkari (2025)	96.0% (best reported)	–	[11, 12%] (95% CI)	–	Train-test split only
Aqachtoul et al. (2025)	100.0% (best reported)	±1.0%	–	–	5-fold
Rishiwal et al. (2025)	100.0% (best reported)	±1.0%	–	–	4-fold

*Statistical Validation Analysis*. Although accuracy was reported by 111 of the 114 reviewed studies (97.4%), only 53 studies (46.5%) reported accuracy together with at least one statistical validation measure, such as standard deviation, confidence intervals, significance testing, or cross-validation. Standard deviation was the most frequently reported statistical validation measure (38.6%), whereas confidence intervals (7.0%) and formal significance testing (19.3%) were comparatively uncommon. These findings indicate that while predictive performance metrics are widely reported, rigorous statistical validation remains inconsistently adopted across the literature. Future research should complement conventional evaluation metrics with appropriate statistical validation to improve the reliability, reproducibility, and comparability of AI-driven IoT intrusion detection systems.

### 14.3 Experimental conditions, assumptions, computational cost, and hardware feasibility

Most of the intrusive studies on AI-enabled IoT security systems have been conducted in a controlled lab environment with publicly available benchmark datasets and a fixed partition between the training and test sets (Sharma and Bairwa, 2025; Cao et al., 2025). While these experimental configurations allow for repeatable testing and benchmarking, they do not necessarily represent the heterogeneous traffic and dynamic attack evolution, intermittent connectivity and resource constraints found in operational IoT and Industrial IoT deployments (Cao et al., 2025). Thus, reported performance is subject to interpretation when scaled up to real world environments (Sharma and Bairwa, 2025).

There are also some simplifying assumptions in the surveyed literature. Typical machine learning and deep learning models typically assume that the dataset is balanced, network connectivity is stable, and a significant amount of computational power is available during training and prediction (Cao et al., 2025). Federated learning methods often require clients to participate honestly, the communication to be reliable, and the models to be aggregated synchronously (Vyas et al., 2024) while TinyML implementations typically rely on stable hardware and known energy availability (Ramadan et al., 2025). These assumptions are useful to simplify experimental evaluation but are not necessarily valid for large scale heterogeneous IoT systems (Sharma and Bairwa, 2025).

Lightweight machine learning and TinyML architectures are characterized by having the smallest computational footprint and are therefore suitable to be deployed on resource-constrained devices like Raspberry Pi, ESP32, STM32 and ARM Cortex based microcontroller (Ramadan et al., 2025). Unlike deep learning architectures, federated learning has its own communication overhead for collaborative optimization, while deep learning architectures typically need GPU acceleration and more memory. The additional computational complexity is also introduced by consensus operations and maintaining a distributed ledger in the case of blockchain-assisted intrusion detection (Ali et al., 2024). As such, there should be an appropriate trade-off between the detection capability of the IDS and the computational resource and deployment requirements (Sharma and Bairwa, 2025).

### 14.4 Adversarial robustness and reproducibility

There is limited research in the literature regarding adversarial robustness (Sharma and Bairwa, 2025; Aparcana-Tasayco et al., 2025). Compared with other studies, the majority of machine learning and deep learning models have been designed for trusted training environments and tested against conventional attacks, with fewer efforts dedicated to studying of poisoning attacks, evasion attacks, model inversion, gradient leakage, membership inference and Byzantine attacks (Vyas et al., 2024; Alfadhil et al., 2025). Federated learning and blockchain-based approaches enhance robustness by securely aggregating data, implementing decentralized trust management systems, and employing privacy-preserving techniques. However, these methods are still susceptible to advanced attacks using adversarial tactics which need further exploration (Ali et al., 2024; Vyas et al., 2024).

Reproducibility is also a key issue in IoT intrusion detection research based on AI (Sharma and Bairwa, 2025; Aparcana-Tasayco et al., 2025). While a large number of studies use other benchmark datasets that are publicly available, only a few of them include complete source code, trained models, preprocessing techniques, hyperparameter settings and deployment scripts. The absence of implementation transparency reduces the possibility of independent validation and makes it difficult to make a fair comparison between different paradigms of intrusion detection (Aparcana-Tasayco et al., 2025). Future work should therefore focus on open-source implementations, standardized benchmarking protocols and reproducible experimental workflows, to enhance the reliability and comparison of published results (Sharma and Bairwa, 2025; Aparcana-Tasayco et al., 2025).

### 14.5 Industrial deployment readiness

Although numerous studies have stated the accuracy of their methods is above 95%, very few have tested their methods with actual IoT hardware such as Raspberry Pi, NVIDIA Jetson Nano, ESP32, STM32 and ARM Cortex devices. The vast majority of the evaluations are limited to simulation environment and publicly available datasets (Sharma and Bairwa, 2025; Aparcana-Tasayco et al., 2025). Although a few examples of promising experimental results have been produced (Sharma and Bairwa, 2025), it remains unknown if many of the proposed intrusion detection systems are industrial ready. For future research, long-term field validation, interoperability of different devices and on standardized deployment benchmarks for Industrial IoT applications should be investigated (Shahin et al., 2024; Aparcana-Tasayco et al., 2025).

In general, lightweight machine learning models are more efficient in computation but heavily rely on manual feature engineering (Nedungadi et al., 2025). Deep learning offers higher detection ability but also has significant computational costs (Jamshidi et al., 2025). The federated learning approach enhances privacy but adds complexities with communication in non-IID settings (Vyas et al., 2024; Alfadhil et al., 2025). TinyML allows real-time on-device intelligence, but is limited by memory and energy (Ramadan et al., 2025). Blockchain enhances trust and integrity, but it also adds latency and storage requirements (Ali et al., 2024). So, no single paradigm can achieve all of these goals – accuracy, scaling, privacy, computational efficiency and adversarial robustness—at the same time (Sharma and Bairwa, 2025). Future IoT intrusion detection systems (IDS) will be using hybrid architectures that combine complementary strengths in each paradigm (Sharma and Bairwa, 2025; Aparcana-Tasayco et al., 2025).

Table 13 summarizes the benchmark datasets, evaluation metrics, representative attack scenarios, computational requirements, practical feasibility of deploying the methods on real hardware, adversarial robustness, maturity of the methods for deployment in industry, and major limitations of the methods reviewed, to facilitate a thorough comparison of the main AI-based paradigms for intrusion detection. This cross-paradigm comparison exposes practical aspects of the different IDS paradigms and offers a unified view of the selection of the proper techniques for various IoT and Industrial IoT environments.

**Table 13:** Cross-paradigm critical comparison of AI-driven IoT intrusion detection approaches with respect to benchmark datasets, evaluation metrics, attack scenarios, computational cost, hardware feasibility, adversarial robustness, industrial deployment maturity, and key limitations.

Paradigm	Common Benchmark datasets	Common evaluation metrics	Primary attack scenarios	Computational cost	Feasibility on real hardware	Adversarial robustness	Industrial deployment maturity	Key limitations
Lightweight machine learning	ToN-IoT, CICIDS2017, BoT-IoT	Accuracy, Precision, Recall, F1-score	DDoS, Botnet, Malware	Low	High (Raspberry Pi, Edge Gateway, ARM Cortex)	Moderate	High	Manual feature engineering; limited adaptability to unknown attacks
Deep learning	CICIDS2017, CICIoT2023, UNSW-NB15	Accuracy, F1-score, MCC, ROC-AUC	Multi-class attacks, Zero-day, Malware	High	Moderate (Jetson Nano, GPU-enabled Edge)	Moderate	Moderate	High computational and memory requirements
Federated learning	ToN-IoT, CICIDS2017, CICIoT2023	Accuracy, Precision, Recall, F1-score	Distributed attacks, Privacy-preserving IDS	Moderate	Moderate (Edge–Cloud Collaborative Platforms)	High (with secure aggregation)	High	Communication overhead; non-IID data challenges
TinyML	ToN-IoT, BoT-IoT	Accuracy, F1-score, Latency, Energy Consumption	Lightweight malware, Device-level intrusion	Very Low	Very High (ESP32, STM32, Arduino Nano, Cortex-M)	Low–Moderate	Moderate	Limited memory, reduced model complexity
Edge–Fog–Cloud	ToN-IoT, CICIDS2017, MQTTEEB-D	Accuracy, Latency, Throughput, QoS	Distributed network attacks, multi-stage attacks	Moderate	High (Edge Servers, Fog Nodes)	Moderate	High	Complex resource orchestration
Blockchain-based IDS	ToN-IoT, IIoT datasets	Accuracy, F1-score, Transaction Latency	Model poisoning, Trust attacks, Tampering	High	Moderate	High	Moderate	Consensus latency, storage overhead
Trust-aware IDS	ToN-IoT, WSN datasets	Accuracy, Trust Score, F1-score	Insider attacks, Compromised devices	Moderate	High	High	Emerging	Dynamic trust management complexity
XAI-based IDS	CICIDS2017, ToN-IoT	Accuracy, SHAP, LIME, Explainability Score	AI-assisted intrusion detection	Moderate–High	Moderate	Moderate	Emerging	Increased computational overhead for explanations

### 14.6 Overall critical synthesis

As shown in Sections 13.1–13.5 and in Table 7, it is evident that while every AI-based paradigm for IDS has its own advantages and disadvantages, none can guarantee that it provides the best detection performance, computational efficiency, privacy preservation, scalability, adversarial robustness, and deployment feasibility all at once (Sharma and Bairwa, 2025; Aparcana-Tasayco et al., 2025). Although lightweight models are appealing to resource-limited IoT devices as they are lightweight and can be readily executed on embedded hardware, they are vulnerable to the requirement of manual feature engineering, which can be a hindrance in adapting to previously unseen attack patterns (Nedungadi et al., 2025; Bekkouche et al., 2022). Deep learning architectures have shown consistently high detection performance and feature representation on benchmark datasets for the use of considerable computational resources, memory capacity, and GPU acceleration, thus making it difficult to deploy on a low-power edge device (Jamshidi et al., 2025).

Federated learning is a suitable approach in distributed IoT settings as it is designed to alleviate privacy concerns by facilitating collaborative model training without centralized data collection. However, the issues of communication overhead, client heterogeneity, non-IID data distributions and susceptibility to poisoning attacks are still major issues in deployment (Vyas et al., 2024; Alfadhil et al., 2025). While TinyML takes intelligent intrusion detection right into the microcontroller and embedded device via model compression and light-weight inference, the capability to detect complicated or evolving cyberattacks could be limited by the memory, energy and processing constraints of these devices (Ramadan et al., 2025). Likewise, collaborative architectures based on the Edge-Fog-Cloud paradigm enhance scalability and real-time responsiveness by breaking this up into multiple layers, yet the challenges of orchestration, synchronization overhead, and managing heterogeneous infrastructure are still open research issues (Shruti et al., 2024). Blockchain-based intrusion detection enhances trust, integrity and auditability by leveraging decentralized consensus mechanisms, while also adding more latency, storage and consensus overhead which could limit large scale deployments (Ali et al., 2024; Sathyabama and Katiravan, 2025).

Comparative evaluation additionally shows that most published work uses publicly available benchmark datasets, offline experimental setups, and traditional performance measures like accuracy and F1-score, while few studies compare the inference latency, energy consumption, repeatability, adversarial robustness, or long-term deployment on operational Industrial IoT infrastructures (Sharma and Bairwa, 2025; Aparcana-Tasayco et al., 2025). Therefore, it is hard to compare the results of different intrusion detection paradigms directly, due to different data sets, evaluation protocols, and reporting formats (Aparcana-Tasayco et al., 2025).

Overall, the literature indicates that future AI-driven intrusion detection systems are likely to adopt hybrid architectures that integrate complementary strengths from lightweight machine learning, deep learning, federated learning, TinyML, Edge–Fog–Cloud computing, blockchain, trust-aware intelligence, and explainable AI. Future research should therefore emphasize standardized benchmarking, reproducible experimental protocols, comprehensive adversarial evaluation, and large-scale real-world validation to facilitate the deployment of robust, scalable, and trustworthy intrusion detection systems for next-generation IoT and Industrial IoT environments (Sharma and Bairwa, 2025; Aparcana-Tasayco et al., 2025).

## 15 Unified taxonomy of IoT security mechanisms

The literature presented in the current study shows that the modern IoT cybersecurity frameworks have a multi-layered architecture. Modern systems combine various security technologies in various layers of the architecture, rather than having a single isolated detection mechanism. This multi-layered point of view is supported by a network intrusion detection survey across both IoT and non-IoT settings (Kushwaha et al., 2025).

### 15.1 Intelligence mechanisms

The intelligence layer consists of machine learning models, deep learning systems, anomaly detectors and generative models that detect malicious activity (Ozdogan, 2024). A survey of machine learning-based malware detection methods in the context of an IoT setting classifies these intelligence methods according to detection strategy and computational cost (Lefoane et al., 2025).

Intelligence mechanisms are projected to distributed settings with federated learning frameworks (Khan et al., 2024). A detailed literature review of machine learning and deep learning methods of detecting malware in Android and IoT devices also records the scope of intelligence methods that can be used (Ruiz-Guirola et al., 2025).

### 15.2 Optimization and lightweight strategies

Optimization strategies are also very important in facilitating the effectiveness of intrusion detection systems in resource-constrained IoT environments. Some of the typical methods of optimization are feature selection methods, dimensionality reduction methods, model pruning methods and parameter compression methods (Devi et al., 2025). In a systematic review of anomaly detection in IoT security surveys, quantum and classical optimization methods are considered to improve the detection efficiency (Alazab et al., 2023). The variety of optimization strategies used in botnet detection paradigms are recorded in Internet of Things botnet surveys on artificial intelligence-based detection techniques (Sathyabama and Katiravan, 2025). AI malware detection in IoT networks in smart cities gives an overview of optimization techniques used in deploying systems at the urban scale (Ozdogan, 2024).

### 15.3 Deployment architectures

Deployment architectures dictate where to run intrusion detection models. Architectures that are hierarchical with a device, edge, fog and cloud layer are more scalable and have lower detection latency (Rey et al., 2022). Layered deployment in device and edge layers is shown by hierarchical lightweight intrusion detection using deep learning in the context of IoT (Akshya et al., 2025). Further development of IoT security by performing a systematic review of machine learning-based methods of botnet detection classifies deployment architectures based on communication topology and detection layer (Pandey et al., 2025). Proactive deployment strategies identified by AI-assisted prediction of attack precursors based on a machine learning approach to predictive cybersecurity illustrate proactive deployment techniques that identify attacks before they happen (Guizani and Ghafoor, 2020).

### 15.4 Adaptive defense mechanisms

The highest level of IoT cybersecurity structures is adaptive defense mechanisms. Adaptive defense systems alter system configurations to interrupt cyberattacks rather than relying on detection alone. Moving Target Defense (MTD) systems dynamically vary network parameters, including IP addresses, runtime libraries and system settings, to decrease the predictability of attacks. A lightweight Moving Target Defense system for multi-purpose IoT malware shows that MTD is a better way of making sustained attacks more expensive (Von Der Assen et al., n.d.). AI-powered malware detectors for monitoring industrial IoT represent another dynamic solution where detection models are retrained according to new threat intelligence (Smmarwar et al., 2023).

## 16 Mathematical foundations of lightweight FL-ids for IOT

While the preceding sections critically reviewed machine learning, deep learning, federated learning, TinyML, edge computing, blockchain, and explainable AI techniques for IoT intrusion detection, this section consolidates the key mathematical formulations that repeatedly appear across the surveyed literature (Sharma and Bairwa, 2025; Aparcana-Tasayco et al., 2025). The objective is not to introduce new algorithms, but to provide the theoretical foundations that explain how lightweight federated intrusion detection systems perform model aggregation, client selection, performance evaluation, and resource-aware deployment (Vyas et al., 2024; Ramadan et al., 2025). The selected mathematical models where those that are most commonly used in the latest FL-based IDS research as well as they offer a common ground for comparison of the various methodologies used in this review throughout (Vyas et al., 2024; Sharma and Bairwa, 2025).

The mathematical formulations introduced in this section directly aid the proposed taxonomy introduced in this review. In particular, the federated aggregation equations refer to the Federated Learning layer (Vyas et al., 2024), the reputation-based client selection model addresses adaptive intelligence under heterogeneous and non-IID IoT environments (Alfadhil et al., 2025), the IDS evaluation metrics provide a standardized framework for comparing ML, DL, FL, and TinyML-based detection models (Sharma and Bairwa, 2025), while the energy and computational analysis support the Lightweight Intelligence and TinyML deployment layers. Therefore, this section provides the theoretical basis that links the conceptual taxonomy with the practical implementation of intelligent IoT intrusion detection systems.

Accordingly, the mathematical formulations presented in this section should be interpreted as analytical abstractions of the machine learning, deep learning, federated learning, TinyML, blockchain, and edge intelligence paradigms reviewed in Sections 4–14 rather than as independent algorithmic contributions.

### 16.1 Federated learning weighted aggregation

In federated learning-based intrusion detection, each participating IoT client i trains a local model on its private dataset D_i_ of size TS_i_ and the global model is built by aggregating local model weights without exposing raw traffic data (Ahmad and Shah, 2024). Formalize the weighted aggregation as:

$$W^{\prime }=\sum_{i=1}^{n}\frac{\mathrm{TSi}}{\mathrm{TS}}\;\mathrm{Wi},$$ (1)

Where W′ is the aggregated global model weight, Wi is the local model weight of client i, TS_i_ is the local training dataset size of network i and TS = $\sum_{i=1}^{n}\mathrm{TSi}$ , is the total dataset size across all participating clients (Ahmad and Shah, 2024). This proportional weighting ensures that clients with larger local datasets contribute more to the global model, reducing the influence of sparse or noisy local datasets. After global aggregation, each edge/fog device updates its local model weights for the next federation round by incorporating the momentum of the weight change between consecutive rounds (Ahmad and Shah, 2024).

$$\mathrm{Wt}+1=W^{\prime }+\lambda (\mathrm{Wt}-W^{\prime }),$$ (2)

Where W_t_ is the local model weight at the previous round and λ ∈ [0,1] is a momentum parameter that controls the contribution of the inter-round weight change. As demonstrated on the TON-IoT network dataset, setting λ = 0.25 reduces the number of required federation rounds while maintaining 98.85% attack detection accuracy with a false alarm rate of only 0.09% (Ahmad and Shah, 2024).

The weighted aggregation model was chosen because most federated learning-based intrusion detection systems, reviewed in this paper, are based on it. The formulation assumes that the participating IoT devices will truthfully report their model parameters and that the size of the local data set is better representative of the updates. This is a general rule in collaborative learning situations but not always true in adversarial or very heterogeneous situations. While weighted aggregation can help maintain data privacy and minimize the amount of data shared centrally, it is vulnerable to non-IID data distributions, client drift, communication delays, and model poisoning attacks. This formulation has been extended in several recent works to adaptive aggregation, trust-aware optimization, and robust federated optimization algorithms to enhance convergence and robustness in realistic deployments of IoT.

*Applicability*: This aggregation model is particularly suitable for privacy-preserving federated intrusion detection deployed across edge, fog, and cloud-assisted IoT environments where raw traffic data cannot be centrally collected.

### 16.2 Reputation—based client selection for non-IID IoT FL

One of the major challenges highlighted during this review is the heterogeneous and non-IID IoT environment and reputation-based client selection tackles this challenge. The mathematical model supposes that client reliability can be estimated based on the historical performance, quality of communication and consistency of the gradient. These assumptions allow for a more powerful participant selection than the conventional FedAvg, but with reputation calculation comes extra computational and communication burden. Moreover, a combination of more advanced attacks that lead to poisoning and malicious clients that attempt to manipulate reputation scores suggest that in the future, federated IDS should include reputation mechanisms with trust management, blockchain verification or secure aggregation techniques.

A fundamental limitation of standard FL in heterogeneous IoT environments is that dominant clients with non-representative local data distributions can bias the global model. To mitigate this limitation, recent studies have proposed a multi-metric reputation scoring system that quantifies the trust worthiness and contribution quality of each FL client (Alfadhil et al., 2025). The five-component metrics are defined as follows.

*Accuracy* (A_i_) measures how well a client’s local model generalizes beyond its own data (Alfadhil et al., 2025).

$$\mathrm{Ai}=\frac{\;\text{Correct Predictions}\;\mathrm{by}\;\text{Client}\;i}{\text{Total Predictions in Global Validation}\;\mathrm{Set}\;}$$ (3)

*Consistency* (C_i_) penalizes clients with unstable contributions across training rounds (Alfadhil et al., 2025).

$$\mathrm{Ci}=1-\frac{\sigma (A_{i,1},A_{i,2,\ldots \ldots \ldots }A_{i,t})}{\mu (A_{i,1},A_{i,2,\ldots \ldots \ldots }A_{i,t})}$$ (4)

Where σ and μ denote the standard deviation and mean of client i’s round-wise accuracy over t rounds. Clients with fluctuating accuracy receive lower C_i_ scores, reducing their weight in aggregation.

*Network condition* (N_i_) prioritizes clients with reliable communication channels (Alfadhil et al., 2025).

$$Ni=\frac{\text{SNRi}}{\text{maxj}\in K(\text{SNRj})}$$ (5)

Where SNR_i_ is the measured signal-to-noise ratio of client i’s transmission channel. Data quality (D_i_) measures the divergence between a client’s gradient and the global gradient, detecting non-IID drift (Alfadhil et al., 2025).

$$\mathrm{Di}=1-\frac{\nabla \mathrm{Fw}(t-1)-\nabla \mathrm{Fiw}(t-1)}{\text{maxj}\in K(\text{Divergencej})}$$ (6)

A client whose local gradient deviates strongly from the global gradient, a direct consequence of non-IID data receives a low D_i_ score and is deprioritized in the aggregation round. Historical reliability (H_i_) combines a moving average of recent accuracy with a trend ratio to capture long-term contribution quality (Alfadhil et al., 2025).

$$\mathrm{Hi}=W_{\mathrm{MA}}\frac{1}{k}\sum_{j=t-k+1}^{t}\mathrm{Ai}(j)+W_{T}\frac{\frac{1}{k}\sum_{j=t-k+1}^{t}\mathrm{Ai}(j)}{\frac{1}{k}\sum_{j=t-2k+1}^{t-k}\mathrm{Ai}(j)}$$ (7)

Where k is the moving-window size and W_MA_ + W_T_ = 1. Finally, the Combined Selection Score (CS_i_) balances reputation quality with proportional fairness (Alfadhil et al., 2025).

$$\mathrm{CSi}=\alpha \;\text{Norm}-\text{Reputatio}n_{i}+(1-\alpha )\text{Norm}-\text{Fairnes}s_{i}$$ (8)

Where α ∈ [0,1] is a tunable parameter: α → 1 prioritizes high-performing clients for rapid convergence, while α → 0 ensures equitable participation of under-represented IoT devices. Evaluated on the UNSW-NB15 dataset under both IID and non-IID conditions, this mechanism significantly reduces the communication rounds needed to reach stable accuracy relative to FedAvg and Fed Prox (Alfadhil et al., 2025).

The reputation-based client selection model was chosen as it solves one of the most critical problems identified during this review: Client heterogeneity and non-IID data distributions in a federated IoT environment. *Assumptions*: It is assumed that the reliability of the client can be measured based on their performance in the past, quality of communication and consistency of gradient. *Applicability*: It is especially suited for large scale deployments of heterogeneous IoT devices that require collaborative learning, where the local data distributions and the computational capacity of the participating devices vary. *Limitations:* Computational and communication overhead from reputation calculation; advanced clients and malicious clients could be able to influence reputation score. Therefore, future federated IDS should combine the reputation mechanism, trust management mechanism, blockchain verification mechanism, and secure aggregation mechanism to enhance the robustness of federated IDS systems.

### 16.3 IDS detection performance metrics

Accuracy, Precision, Recall, and F1-score remain the most frequently reported evaluation metrics across the surveyed IoT IDS literature because they enable consistent comparison of detection performance. However, these metrics assume correctly labelled datasets and balanced attack distributions. In highly imbalanced intrusion datasets, overall accuracy alone may produce misleading conclusions. Therefore, future evaluations should also report communication overhead, inference latency, memory consumption, energy efficiency, Matthews Correlation Coefficient (MCC), ROC-AUC, and robustness against adversarial attacks to provide a more comprehensive assessment of lightweight IoT intrusion detection systems.

Define the standard evaluation metrics used throughout the surveyed IoT IDS literature. Let TP, FP and FN denote true positives, false positives and false negatives, respectively (S’aez-de-C’amara et al., 2023). Accuracy quantifies the fraction of correctly classified traffic instances (S’aez-de-C’amara et al., 2023).

$$\mathrm{Ac}=\frac{\text{Correct Predictions}}{\text{Total Predictions}}$$ (9)

*Precision* measures the reliability of positive (attack) predictions (S’aez-de-C’amara et al., 2023).

$$\mathrm{Pr}=\frac{\mathrm{TP}}{\mathrm{TP}+\mathrm{FP}},$$ (10)

*Recall* (Detection Rate) captures the fraction of actual attacks correctly identified (S’aez-de-C’amara et al., 2023).

Re=TPTP+FN (11)

*F1-Score* harmonizes precision and recall into a single IDS quality measure (S’aez-de-C’amara et al., 2023).

F1=2∗Pr∗RePr+Re (12)

Applying these metrics, the mFCBF-based lightweight IDS achieves 99.75% accuracy on the BoT-IoT dataset while consuming only 2.03% average CPU and 1.2% memory, confirming that lightweight feature selection preserves detection quality within IoT resource constraints (S’aez-de-C’amara et al., 2023). The stacked ensemble model. Similarly, it reports accuracy, precision, recall and F1 of 99.3, 99.2, 99.0 and 99.1%, respectively on the UNSW BoT-IoT dataset, requiring at least 36% less CPU and 38% less memory than competing methods (Esmaeilyfard et al., 2025).

The evaluation metrics presented above were selected because they represent the most widely adopted performance measures across the lightweight ML, DL, FL, and TinyML intrusion detection studies surveyed in this review. *Assumptions*: These metrics assume correctly labelled datasets and representative class distributions. *Applicability*: They provide a standardized framework for comparing intrusion detection performance across heterogeneous IoT datasets and deployment scenarios. *Limitations*: Accuracy alone may be misleading for imbalanced datasets; therefore, future studies should additionally report MCC, ROC-AUC, communication overhead, inference latency, energy consumption, and adversarial robustness for comprehensive evaluation.

### 16.4 Energy and computational cost in IoT IDS

The ability to identify the resources of an intrusion detection system is particularly relevant for lightweight IoT intrusion detection systems, as they must work on different deployment platforms that have very different processing power, memory space, battery life and communication bandwidth. Discussed energy models make generic assumptions about hardware configurations and workload; reported energy consumption may differ depending on the IoT device. Various studies clearly show a compromise between detection accuracy, computational cost, communication overhead and energy usage, meaning that future lightweight IDS should try to balance these parameters, rather than optimise for a single one.

Conduct the first empirical study evaluating the joint impact of ML-based IDS on CPU usage, energy consumption and CPU load at the edge of IoT infrastructures. Their key finding is that traditional ML-based IDS, when deployed at the edge gateway, significantly increases energy consumption and CPU load during real-time cyber threat scenarios, while deep learning-based IDS incurs even higher resource overhead. This creates a measurable three-way tension between detection accuracy, computational cost and energy budget that must be addressed in any practical deployment (Jamshidi et al., 2025).

Further demonstrate that energy-based side-channel monitoring- leveraging CPU power profiles, RAM usage and electromagnetic signatures-can itself serve as a lightweight attack detection mechanism, complementing network traffic-based IDS without requiring dedicated inference hardware. The survey identifies that deviations from baseline energy consumption patterns reliably indicate botnet, DDoS and malware activity in IoT devices (Merlino and Allegra, 2024). Quantify the energy profile of TinyML deployments. Typical microcontroller-based IoT clients operate at milliwatts to microwatts, which is three orders of magnitude below conventional CPUs (up to 90 W) and GPUs (200–600 W). Model compression techniques, including quantisation, pruning and run-length encoding- reduce transmission cost and energy per inference while introducing an accuracy trade-off that must be characterized per attack class in security-critical deployments (Ramadan et al., 2025).

The energy and computational models discussed above were selected because resource efficiency represents one of the primary design constraints of lightweight IoT intrusion detection systems. *Assumptions*: The reported resource consumption assumes representative hardware configurations and comparable deployment environments. *Applicability*: These models are directly applicable to TinyML-enabled IoT devices, edge gateways, and battery-powered embedded systems where computational resources are limited. *Limitations*: Energy consumption varies across hardware platforms, communication technologies, and optimization techniques. Future research should therefore develop unified optimization frameworks that jointly consider detection accuracy, communication efficiency, latency, and energy consumption.

### 16.5 Mathematical insights and future research directions

The mathematical models outlined in this section suggest numerous avenues for future research of lightweight federated intrusion detection systems. Current aggregation models focus on maximizing the detection accuracy, but suffer from low robustness to extreme non-IID data distribution, adversarial clients and communication limitations. Further study is needed on adaptive optimization algorithms that can address all three challenges of client heterogeneity, trust management, and communication efficiency. Likewise, energy, inference latency, communication cost, explainability and robustness should be treated as first-class optimization goals, in addition to classification performance, in the evaluation metrics. Mathematical optimization of model compression, quantization, pruning and adaptive inference is still an open research area for TinyML-enabled IoT devices. The opportunities directly correspond to the proposed taxonomy and underscore the importance of single-architecture optimization methods that are able to achieve high detection accuracy, preserve user privacy, ensure computational efficiency, and facilitate sustainable deployment in next-generation IoT and IIoT.

## 17 Research gap analysis and open problems

These gaps represent problems that (i) are confirmed absent from the existing literature, (ii) can be addressed using current datasets such as ToN-IoT and CICIoT and (iii) are implementable within a 6–12 months research timeline using available tools such as TensorFlow Federated, Flower and TensorFlow Lite. These gaps represent critical limitations in existing IoT intrusion detection approaches, particularly in balancing accuracy, privacy, scalability and resource efficiency, as comparatively illustrated in Figure 10.

**Figure 10:**
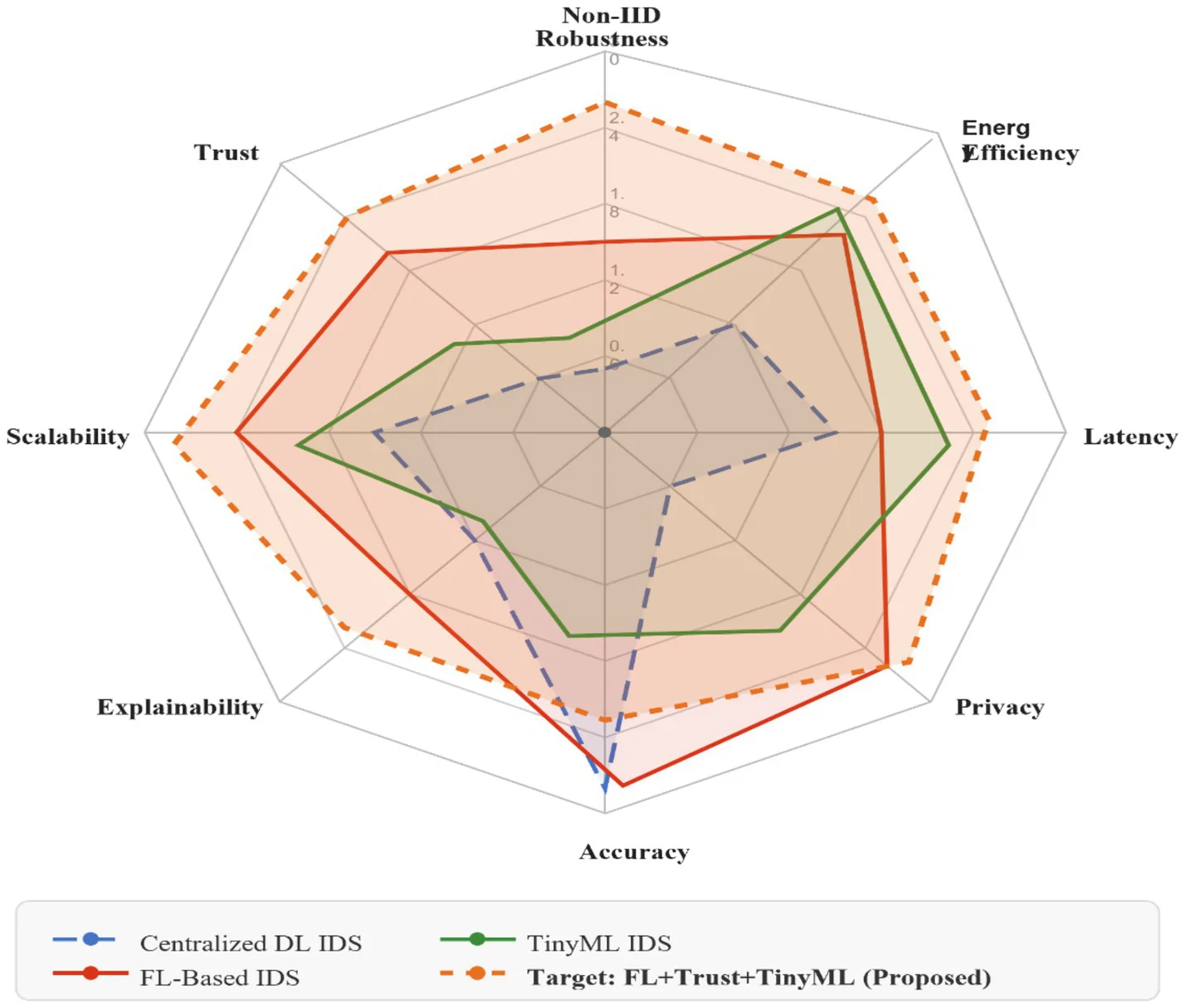
Research gap radar has eight IDS evaluation dimensions. Grey areas between existing approaches (solid/dashed polygons) and the target envelope (orange dotted) represent open research problems. Non-IID Robustness and Explainability show the largest gaps across all existing paradigms (created by the authors based on the reviewed literature).

### 17.1 Gap 1: non-IID convergence under real-time latency constraints

Existing federated learning solutions for IoT intrusion detection address non-IID heterogeneity and real-time latency independently but never simultaneously. Clustered FL groups devices by data similarity to reduce client drift, yet the clustering overhead adds several seconds of computation per federation round to a latency budget that is incompatible with sub-100 ms intrusion response requirements in industrial IoT settings. Reputation-based aggregation accelerates convergence under non-IID conditions but requires a warm-up period of 10–20 rounds before stable reputation scores are established, during which the global model remains blind to device-specific traffic patterns.

The gap is therefore that no existing FL-IDS architecture co-optimizes convergence speed under non-IID data distributions and real-time detection latency simultaneously on heterogeneous IoT hardware.

### 17.2 Gap 2: protocol-agnostic IDS generalization

Every major IoT security dataset in the surveyed literature, ToN-IoT, MQTTEEB-D, IoT Z-Wave, MTC Statistical Dataset, is protocol-specific. Models trained on ToN-IoT (HTTP, Domain Name System (DNS), MQTT traffic) fail to generalize to Z-Wave or Modbus environments without full retraining. This fragmentation forces practitioners to maintain separate IDS models for each IoT communication protocol, multiplying deployment complexity and resource overhead.

The gap is that no single lightweight IDS model has been demonstrated to achieve over 90% detection rate simultaneously across three or more IoT communication protocols without protocol-specific fine-tuning.

### 17.3 Gap 3: cold-start problem in trust-aware federated IDS

Reputation-based FL and trust-aware IDS frameworks both depend on accumulated interaction history to score device trustworthiness. However, the rapidly growing IoT ecosystem continuously introduces new, previously unseen devices that have no reputation history. Current systems either assign new devices a neutral default score (causing them to be immediately exploitable as attack vectors) or exclude them entirely from the federation (reducing model diversity and coverage).

The gap is that no trust-aware FL-IDS framework provides a validated mechanism for bootstrapping reputation scores for newly joining IoT devices without requiring prior interaction history.

### 17.4 Gap 4: TinyML detection floor for security-critical attack classes

TinyML research demonstrates significant energy and memory savings from quantization and pruning but evaluates accuracy at the aggregate level. For IoT IDS, the critical figure of merit is not overall accuracy but the detection rate on the most dangerous and low-frequency attack classes- specifically slow-rate DDoS, insider threats and firmware injection. Which constitute fewer than 0.5% of traffic samples in benchmark datasets yet represent the highest operational risk.

The gap is that no TinyML-based IDS paper quantifies the per-attack-class detection rate under INT4 or INT8 quantization for low-frequency, high-severity attack categories on real IoT hardware.

### 17.5 Gap 5: adversarial robustness under combined non-IID and poisoning condition

Label-flipping and model-poisoning adversarial attacks against FL-IDS have been studied exclusively in IID or near-IID settings. Real IoT deployments are inherently non-IID and the interaction between non-IID data heterogeneity and adversarial poisoning is uncharacterized. In non-IID settings, the natural gradient divergence between honest clients may mask the anomalous gradients of adversarial clients, making standard Byzantine-robust aggregation methods (e.g., Krum, coordinate-wise median) unreliable.

The gap is that no existing work evaluates FL-IDS adversarial robustness specifically under non-IID + simultaneous label flipping conditions in IoT network traffic distributions.

### 17.6 Gap 6: lightweight Explainability for edge-deployed IDS

Explainable AI methods applied to IoT IDS in the surveyed literature, including SHAP and LIME, are exclusively post-hoc methods that require full model access and multiple inference passes. Their computational cost (typically 10–100 inference equivalents per explanation) is prohibitive on microcontroller-class IoT hardware and even on edge gateways with constrained RAM. Consequently, no existing IoT IDS deployment at the device or edge level provides real-time, human-interpretable explanations alongside detection alerts.

The gap is that no lightweight, on-device XAI mechanism exists for IoT IDS that produces interpretable feature attribution at inference time within the energy and latency budget of a microcontroller or edge gateway.

## 18 Limitations and future research directions

### 18.1 Limitations of this review

1 Limited Literature Scope: This review includes only Scopus-indexed, peer-reviewed journal articles published between 2020 and 2026, excluding conference papers, book chapters, non-English publications, and studies from other databases.
2 Heterogeneous Experimental Settings: Differences in datasets, attack scenarios, evaluation metrics, feature engineering, and hardware platforms limit direct comparison among the reviewed studies.
3 Limited Statistical Validation: Many studies reported detection accuracy but lacked comprehensive statistical validation, such as confidence intervals, significance testing, or repeated cross-validation.
4 Benchmark Dataset Dependency: Most studies relied on public benchmark datasets, with relatively few validating their methods in real-world IoT or Industrial IoT environments.
5 Deployment Constraints: Several studies did not comprehensively evaluate computational complexity, energy consumption, memory usage, communication overhead, or long-term deployment feasibility.

### 18.2 Future research directions

1 Standardized Benchmarking: Develop unified benchmarking frameworks with standardized datasets, evaluation metrics, and statistical validation protocols.
2 Real-World Deployment: Validate intrusion detection systems in operational IoT and Industrial IoT environments across diverse application domains.
3 Lightweight and Energy-Efficient AI: Advance TinyML and lightweight AI models through compression, pruning, and hardware-aware optimization.
4 Trustworthy Federated Learning: Improve Federated Learning by addressing non-IID data, communication overhead, secure aggregation, and adversarial attacks.
5 Blockchain-Enabled Security: Develop scalable and lightweight blockchain mechanisms for secure aggregation, authentication, and trust management.
6 Explainable AI (XAI): Design lightweight and interpretable XAI techniques that improve transparency without compromising detection performance.
7 Adversarially Robust IDS: Develop intrusion detection systems resilient to adversarial attacks, including model poisoning and evasion attacks.
8 Foundation Models and LLMs: Investigate the use of foundation models and LLMs for intelligent threat detection and automated cybersecurity analysis.
9 Quantum-Resistant Security: Explore post-quantum cryptographic techniques to strengthen future IoT security architectures.
10 Autonomous Self-Healing Security: Develop self-healing AI-driven security systems capable of adaptive detection, response, and recovery.

## 19 Conclusion

The world of IoT has created a new cybersecurity landscape. The traditional centralized intrusion detectors are not sufficient to provide security in large-scale distributed environments with a wide range of devices, limited computational resources and continuous data inputs. A network intrusion detection survey covering IoT and non-IoT settings and scenarios validates this evaluation.

The study focused on the latest advancements in IoT IDS, such as lightweight machine learning models, deep learning models, federated learning models, TinyML-based edge intelligence, blockchain-based trust management, and explainable artificial intelligence methods and adaptive defense mechanisms.

Efficient real-time monitoring of IoT devices with limited resources is offered by lightweight detection models. In a federated learning system, the models are trained in a distributed network without compromising privacy. Blockchain technologies can enhance trust management and boost the trustworthiness of collaborative security frameworks. Explainable AI techniques improve the interpretability and transparency of machine learning-powered IDSs.

The integration of these technologies into multi-layer security architectures enables scalable and adaptive protection for modern IoT ecosystems. Future research should focus on improving adversarial robustness, optimizing communication efficiency in federated learning systems, developing energy-aware detection models and integrating emerging technologies such as quantum-resistant cryptography and intelligent autonomous defense mechanisms.

Distributed intelligence, lightweight machine learning, and adaptive security architectures will be key enablers for the security of next-generation IoT and Industrial IoT infrastructures in the face of emerging cyber threats.

## References

[ref1] Abdulkareem S. A. Foh C. H. Shojafar M. Carrez F. Moessner K., "Network Intrusion Detection: an IoT and non-IoT Related Survey," (2024). IEEE: New York, NY, USA.

[ref2] Abualghanam O. Alazzam H. Almobaideen W. (2025). Hierarchical lightweight intrusion detection system using deep learning in the context of IoT. Clust. Comput. 28:783. doi: 10.1007/s10586-025-05364-3

[ref3] Ahmad M. S. Shah S. M. (2024). A lightweight mini-batch federated learning approach for attack detection in IoT. Internet Things 25:101088. doi: 10.1016/j.iot.2024.101088

[ref4] Akshya J. Sundarrajan M. Vijayakumar R. Dhanaraj R. K. Nayyar A. (2025). Explainable AI-driven intrusion detection for securing IoT-enabled autonomous transportation systems. Clust. Comput. 28:884. doi: 10.1007/s10586-025-05617-1

[ref5] Al Abdulwahid A. (2025). AI-driven identification of attack precursors: a machine learning approach to predictive cybersecurity. Comput. Mater. Contin. 85, 1751–1777. doi: 10.32604/cmc.2025.066892

[ref6] Al Amro S. (2025). Securing internet of things devices with federated learning: a privacy-preserving approach for distributed intrusion detection. Comput. Mater. Contin. 83, 4623–4658. doi: 10.32604/cmc.2025.063734

[ref7] Alanezi K. Annapareddy T. Khan S. Mishra S. (2026). An edge-based IDS for the IoT using combined ML and generative AI models. Peer-to-Peer Netw. Appl. 19:24. doi: 10.1007/s12083-025-02174-7

[ref8] Alazab A. Khraisat A. Singh S. Jan T. (2023). Enhancing privacy preserving intrusion detection through federated learning. Electronics 12:3382. doi: 10.3390/electronics12163382

[ref9] Alfadhil M. J. Baydoun A. Alazab M. Rehman H. U. Jaam J. Al-Maadeed S. A. S. A. (2025). Enhancing federated learning for IoT-based anomaly detection: a reputation-based client selection approach. Alex. Eng. J. 130, 889–909. doi: 10.1016/j.aej.2025.09.019

[ref10] Alhamdi M. J. M. AI-Based Malware Detection in IoT Networks Within Smart Cities: A Survey (2025). Amsterdam, Netherlands.

[ref11] Ali S. Li Q. Yousafzai A., “Blockchain and Federated Learning-Based Intrusion Detection Approaches for Edge-Enabled Industrial IoT Networks: A Survey” (2024). Amsterdam, Netherlands.

[ref12] Almadhor A. Alsubai S. Kryvinska N. al Hejaili A. Bouallegue B. Ayari M. . (2025). Transfer learning with XAI for robust malware and IoT network security. Sci. Rep. 15:26971. doi: 10.1038/s41598-025-12404-w, 40707655 PMC12289998

[ref13] Almobaideen W. Abu Alghanam O. Abdullah M. Hussain S. B. Alam U. (2025). Comprehensive review on machine learning and deep learning techniques for malware detection in android and IoT devices. Int. J. Inf. Secur. 24. doi: 10.1007/s10207-025-01027-x

[ref14] Alnasser O. Al Muhtadi J. Saleem K. Shrestha S. (2025). Signature and anomaly-based intrusion detection system for secure IoTs and V2G communication. Alex. Eng. J. 125, 424–440. doi: 10.1016/j.aej.2025.03.068

[ref15] Alotaibi J. (2025). AI-driven intrusion detection and mitigation framework for software-defined IoT networks. Peer-to-Peer Netw. Appl. 18:321. doi: 10.1007/s12083-025-02151-0

[ref16] Alrayes F. S. Maray M. Alshuhail A. Almustafa K. M. Darem A. A. al-Sharafi A. M. . (2025). Privacy-preserving approach for IoT networks using statistical learning with optimization algorithm on high-dimensional big data environment. Sci. Rep. 15:3338. doi: 10.1038/s41598-025-87454-1, 39870824 PMC11772597

[ref17] Alsekait D. Zakariah M. Amin S. U. Khan Z. I. Alqurni J. S. (2024). Privacy preservation in IoT devices by detecting obfuscated malware using wide residual network. Comput. Mater. Contin. 81, 2395–2436. doi: 10.32604/cmc.2024.055469

[ref18] Alzubi J. A. Alzubi O. A. Qiqieh I. (2025). A feature-learning-enabled malware analysis for enhanced IoT-centric cybersecurity. Clust. Comput. 28:874. doi: 10.1007/s10586-025-05549-w

[ref19] Anwer R. W. . (2025). TEAD: trust-enhanced anomaly detection framework for intrusion detection in IoT-enabled wireless sensor networks. Wirel. Netw 31, 4179–4197. doi: 10.1007/s11276-025-03987-3

[ref20] Aparcana-Tasayco A. J. Deng X. Park J. H. (2025). A systematic review of anomaly detection in IoT security: towards quantum machine learning approach. EPJ Quantum Technol. 112. doi: 10.1140/epjqt/s40507-025-00414-6

[ref21] Aqachtoul A. Karam K. Elamrani A. Najib M. Rafalia N. Bakhouya M. (2025). MQTTEEB-D: a real-world IoT cybersecurity dataset for AI-powered threat detection in MQTT networks. Data Brief 62:111897. doi: 10.1016/j.dib.2025.111897, 40785732 PMC12332880

[ref22] Baawi S. S. Oleiwi Z. C. Al-Muqarm A. M. A. Al-Shammary D. Sufi F. (2025). Efficient malware detection based on machine learning for enhanced cloud privacy protection. Evol. Syst. 16:30. doi: 10.1007/s12530-025-09661-5

[ref23] Baimukhanov S. Ali H. Yazici A. (2025). Enhancing ML-based anomaly detection in data management for security through integration of IoT, cloud and edge computing. Expert Syst. Appl. 293:128700. doi: 10.1016/j.eswa.2025.128700

[ref24] Bala B. Behal S. (2024). “AI techniques for IoT-based DDoS attack detection,” in Taxonomies, Comprehensive Review and Research Challenges, (Amsterdam, Netherlands).

[ref25] Bekkouche R. “Ultra-lightweight and secure intrusion detection system for massive-IoT networks,” in IEEE ICC (2022) pp. 5719–5724.

[ref26] Bensaid R. Labraoui N. Saidi H. Bany Salameh H. (2025). Securing fog-assisted IoT smart homes: a federated learning-based intrusion detection approach. Clust. Comput. 28:40. doi: 10.1007/s10586-024-04711-0

[ref27] Bhatia K. Bhattacharya S. Sharma I. (2024). Privacy-Preserving Detection of DDoS Attacks in IoT using Federated Learning Techniques. New York, NY, USA.

[ref28] Bhavsar M. H. Bekele Y. B. Roy K. Kelly J. C. Limbrick D. (2024). FL-IDS: federated learning-based intrusion detection system using edge devices for transportation IoT. IEEE Access 12, 52215–52226. doi: 10.1109/ACCESS.2024.3386631

[ref29] Bonsu M. A. Akekudaga P. (2025). Privacy-Preserving Lightweight IDS for smart home Botnet Defense. IEEE: New York, NY, USA.

[ref30] Cao Z. Zhao Z. Shang W. Ai S. Shen S. (2025). Using the ToN-IoT dataset to develop a new intrusion detection system for industrial IoT devices. Multimed. Tools Appl. 84, 16425–16453. doi: 10.1007/s11042-024-19695-7, 30311153

[ref31] Chaganti R. Ravi V. Pham T. D. (2022). Deep learning based cross architecture internet of things malware detection and classification. Comput. Secur. 120:102779. doi: 10.1016/j.cose.2022.102779

[ref32] Chen J. Wu M. Gan W. Huang H. Lau T. S. C. (2025). A lightweight CNN malware classification method for software detection. Int. J. Mach. Learn. Cybern. 16, 7405–7423. doi: 10.1007/s13042-025-02662-5

[ref33] Chen Z. Xia W. Li Z. Xiong G. Gou G. Zhang H. . (2025). HoleMal: a lightweight IoT malware detection framework based on efficient host-level traffic processing. Comput. Secur. 152:104360. doi: 10.1016/j.cose.2025.104360

[ref34] Ciaramella G. Martinelli F. Peluso C. Santone A. Mercaldo F. (2026). A method for real-world privacy-preserving android malware detection through federated machine learning. Inf. Softw. Technol. 189:107892. doi: 10.1016/j.infsof.2025.107892

[ref35] Das S. Singh M. P. Namasudra S. (2024). Lightweight and privacy preserving device-to-device authentication to enable secure transitive communication in IoT-based smart healthcare systems. J. Ambient. Intell. Humaniz. Comput. 15, 3331–3345. doi: 10.1007/s12652-024-04810-1

[ref36] Devi M. Nandal P. Sehrawat H. (2025). Federated learning-enabled lightweight intrusion detection system for wireless sensor networks: a cybersecurity approach against DDoS attacks in smart city environments. Intell. Syst. Appl. 27:200553. doi: 10.1016/j.iswa.2025.200553

[ref37] El-Ghamry A. Gaber T. Mohammed K. K. Hassanien A. E. (2023). Optimized and efficient image-based IoT malware detection method. Electronics 12. doi: 10.3390/electronics12030708

[ref38] Eslamnejad M. Taheri R. Shojafar M. Bader-El-Den M. (2025). Federated learning-based robust android malware detection: label-flipping attacks and defenses. Neural Comput. & Applic. 37, 27057–27082. doi: 10.1007/s00521-025-11656-x

[ref39] Esmaeilyfard R. Shoaei Z. Javidan R. (2025). A lightweight and efficient model for botnet detection in IoT using stacked ensemble learning. Soft. Comput. 29, 89–101. doi: 10.1007/s00500025-10425-1

[ref40] Ferrag M. A. Ndhlovu M. Tihanyi N. Cordeiro L. C. Debbah M. Lestable T. . (2024). Revolutionizing cyber threat detection with large language models: a privacy-preserving BERT-based lightweight model for IoT/IIoT devices. IEEE Access 12, 23733–23750. doi: 10.1109/ACCESS.2024.3363469

[ref41] Ficco M. Guerriero A. Milite E. Palmieri F. Pietrantuono R. Russo S. (2024). Federated learning for IoT devices: enhancing TinyML with on-board training. Inf. Fusion 104. doi: 10.1016/j.inffus.2023.102189

[ref42] Guizani N. Ghafoor A. (2020). A network function virtualization system for detecting malware in large IoT based networks. IEEE J Sel Areas Commun 38, 1218–1228. doi: 10.1109/JSAC.2020.2986618

[ref43] Hakiki R. I. Azerine A. Tlemsani R. Golabi M. Idoumghar L. (2025). Enhancing IoT intrusion detection with genetic algorithm-optimized convolutional neural networks. J. Supercomput. 81. doi: 10.1007/s11227-025-07626-8

[ref44] Hasan T. Tasnim S. (2025). Real-time explainable IoT security with machine learning and CTGAN-enhanced detection for resource constrained devices. Ad Hoc Netw. 178. doi: 10.1016/j.adhoc.2025.103937

[ref45] Hasan N. “IoT Botnet Detection Framework from Network Behavior based on Extreme Learning Machine,” in INFOCOM WKSHPS 2022 (2022).

[ref46] Hendaoui F. Meddeb R. Trabelsi L. Ferchichi A. Ahmed R. (2025). FLADEN: federated learning for anomaly detection in IoT networks. Comput. Secur. 155. doi: 10.1016/j.cose.2025.104446

[ref47] Jamshidi S. Nafi K. W. Nikanjam A. Khomh F. (2025). Evaluating machine learning-driven intrusion detection systems in IoT: performance and energy consumption. Comput. Ind. Eng. 204. doi: 10.1016/j.cie.2025.111103

[ref48] Kalakoti R. N˜omm S. Bahsi H. (2025). Federated learning of explainable AI (FedXAI) for deep learning-based intrusion detection in IoT networks. Comput. Netw. 270. doi: 10.1016/j.comnet.2025.111479

[ref49] Kaliappan C. P. Palaniappan K. Ananthavadivel D. Subramanian U. (2024). Advancing IoT security: a comprehensive AI based trust framework for intrusion detection. Peer-to-Peer Netw. Appl. 17, 2737–2757. doi: 10.1007/s12083-024-01684-0

[ref50] Kamatchi K. Uma E. (2025). Securing the edge: privacy-preserving federated learning for insider threats in IoT networks. J. Supercomput. 81. doi: 10.1007/s11227-02406752-z

[ref51] Karmous N. Jlassi W. Aoueileyine M. O.-E. Filali I. Bouallegue R. (2025). A new dataset for network flooding attacks in SDN-based IoT environments. Comput. Model. Eng. Sci. 145, 4363–4393. doi: 10.32604/cmes.2025.074178

[ref52] Kasarapu S. Shukla S. Dinakarrao S. M. P. (2024). Resource- and workload-aware malware detection through distributed computing in IoT networks. ASP-DAC, 368–373. doi: 10.1109/ASP.DAC58780.2024.10473814

[ref53] Khan S. H. Alahmadi T. J. Ullah W. Iqbal J. Rahim A. Alkahtani H. K. . (2023). A new deep boosted CNN and ensemble learning based IoT malware detection. Comput. Secur. 133. doi: 10.1016/j.cose.2023.103385

[ref54] Khan M. M. Buriro A. Ahmad T. Ullah S. (2024). Backdoor malware detection in industrial IoT using machine learning. Comput. Mater. Contin. 81, 4691–4705. doi: 10.32604/cmc.2024.057648

[ref55] Kharnotia S. Arora B. Kour R., Feature-driven static Analysis for Learning-based android Malware Detection: A Review, (2026).

[ref56] Khraisat A. Alazab A. Alazab M. Obeidat A. Singh S. Jan T. (2025). Federated learning for intrusion detection in IoT environments: a privacy-preserving strategy. Discov. Internet Things 5. doi: 10.1007/s43926-025-00169-7

[ref57] Kirubavathi G. . (2025). Enhancing IoT resilience at the edge: a resource efficient framework for real-time anomaly detection in streaming data. CMES 143, 3005–3031. doi: 10.32604/cmes.2025.065698

[ref58] Kumar C. Chittora P. (2024). Secure and privacy-preserving framework for IoT-enabled smart grid environment. Arab. J. Sci. Eng. 49, 3063–3078. doi: 10.1007/s13369-023-07900-y

[ref59] Kumar A. Shridhar M. Swaminathan S. Lim T. J. (2022). Machine learning-based early detection of IoT botnets using network edge traffic. Comput. Secur. 117. doi: 10.1016/j.cose.2022.102693

[ref60] Kushwaha J. P. Bhadauria S. Tapaswi S. (2025). mFCBF based lightweight intrusion detection system for IoT networks. Clust. Comput. 28. doi: 10.1007/s10586-024-05022-0

[ref61] Lefoane M. Ghafir I. Kabir S. Awan I. U., "Internet of Things Botnets: a Survey on Artificial Intelligence-Based Detection Techniques," (2025).

[ref62] Mabrouk K. A. A Federated Intrusion Detection Framework for IoT using visual Encoding and Lightweight Secure Aggregation, in NILES (2025).

[ref63] Mahmood K. Khan S. Abdelhaq M. Hassan M. U. Uddin M. Alsaqour R. . (2025). Adaptive resource aware and privacy preserving federated edge learning framework for real time internet of medical things applications. Sci. Rep. 15:36468. doi: 10.1038/s41598-025-23398-w, 41116048 PMC12537902

[ref64] Mahmud S. A. Islam N. Islam Z. Rahman Z. Mehedi S. T. (2024). Privacy-preserving federated learning-based intrusion detection technique for cyber-physical systems. Mathematics 12. doi: 10.3390/math12203194

[ref65] Mansour S. Mbugua K. Ogamba H. (2025). IoT Malware Classification using Lightweight Neural Network. IEEE.

[ref66] Mehmmod A. Batool K. Sajid A. Alam M. M. Su’ud M. M. D. Khan I. U. (2025). ERBM: a machine learning-driven rule-based model for intrusion detection in IoT environments. Comput. Mater. Contin. 83, 5155–5179. doi: 10.32604/cmc.2025.062971

[ref67] Merlino V. Allegra D. (2024). Energy-based approach for attack detection in IoT devices: a survey. IoT. doi: 10.1016/j.iot.2024.101306

[ref68] Mittal S. Rajvanshi P. “Towards Lightweight hybrid deep Learning Approach to Malware Detection Enhancement for IoT based Systems,” in IEMEN Tech 2025.

[ref69] Mofidi F. Hounsinou S. G. Bloom G. L-IDS: a multi-layered approach to ransomware detection in IoT, in IEEE CCWC (2024) pp. 387–396.

[ref70] Mohamed H. Koroniotis N. Schiliro F. Moustafa N. (2025). IoT-CAD: a comprehensive digital forensics dataset for AI-based cyberattack attribution detection methods in IoT environments. Ad Hoc Netw. 174. doi: 10.1016/j.adhoc.2025.103840

[ref71] Nawaz M. Tahira S. Shah D. Ali S. Tahir M. (2025). Lightweight machine learning framework for efficient DDoS attack detection in IoT networks. Sci. Rep. 15:24961. doi: 10.1038/s41598-025-10092-0. [40640290 PMC12246053

[ref72] Nawshin F. Unal D. Hammoudeh M. Suganthan P. N. (2024). AI-powered malware detection with differential privacy for zero trust security in internet of things networks. Ad Hoc Netw. 161. doi: 10.1016/j.adhoc.2024.103523

[ref73] Nazir A. Advancing IoT Security: A Systematic Review of Machine Learning Approaches for the Detection of IoT Botnets," (2023).

[ref74] Nedungadi N. Sankaran S. Achuthan K. (2025). Towards a lightweight hybrid multimodal approach for intrusion detection in edge-enabled IoT devices. Clust. Comput. 28. doi: 10.1007/s10586-025-05723-0

[ref75] Ngo Q. D. Nguyen H. T. Le V. H. Nguyen D. H., “A Survey of IoT Malware and Detection Methods Based on Static Features, (2020).

[ref76] Nguyen V. T. Beuran R. (2025). FedMSE: semi-supervised federated learning approach for IoT network intrusion detection. Comput. Secur. 151:104337. doi: 10.1016/j.cose.2025.104337

[ref77] Nobakht M. Javidan R. Pourebrahimi A. (2024). SIM-FED: secure IoT malware detection model with federated learning. Comput. Electr. Eng. 116. doi: 10.1016/j.compeleceng.2024.109139

[ref78] Ozdogan E. (2024). A comprehensive analysis of the machine learning algorithms in IoT IDS systems. IEEE Access 12, 46785–46811. doi: 10.1109/ACCESS.2024.3382539

[ref79] Padmavathi V. Saminathan R. (2025). A federated edge intelligence framework with trust-based access control for secure and privacy preserving IoT systems. Sci. Rep. 15:35832. doi: 10.1038/s41598-025-19712-1, 41087484 PMC12521663

[ref80] Pandey V. K. Sahu D. Prakash S. Rathore R. S. Dixit P. Hunko I. (2025). A lightweight framework to secure IoT devices with limited resources in cloud environments. Sci. Rep. 15:26009. doi: 10.1038/s41598-025-09885-0, 40676045 PMC12271337

[ref81] Patel A. Ghosh S. M. (2025). EML-AMD: an explainable machine learning framework for adaptive android malware detection. Peer-to-Peer Netw. Appl. 18. doi: 10.1007/s12083-025-02069-7

[ref82] Portales S. Z. Riegler M. A. (2024). Maleficent neural networks, the embedding of malware in neural networks: a survey. IEEE Access 12, 69753–69764. doi: 10.1109/ACCESS.2024.3401578

[ref83] Puviarasu A. Sudha V. K. (2026). Enhanced IoT security: privacy preserving federated learning model for accurate, real-time intrusion detection across devices. Ain Shams Eng. J. 17:103866. doi: 10.1016/j.asej.2025.103866

[ref84] Ragab M. Ashary E. B. Alghamdi B. M. Aboalela R. Alsaadi N. Maghrabi L. A. . (2025). Advanced artificial intelligence with federated learning framework for privacy-preserving cyberthreat detection in IoT-assisted sustainable smart cities. Sci. Rep. 15:4470. doi: 10.1038/s41598-025-88843-2, 39915579 PMC11803112

[ref85] Rahmati M. Rahmati N. (2025). Adaptive federated edge intelligence for real-time cyberthreat detection in resource-constrained IoT environments: a lightweight deep learning approach. J. Comput. Virol. Hacking Tech. 21. doi: 10.1007/s11416-025-00582-0

[ref86] Ramadan M. N. A. Federated Learning and TinyML on IoT Edge Devices: Challenges, Advances and Future Directions,” (2025). Beijing, China.

[ref87] Rampone G. Ivaniv T. Rampone S. (2025). A hybrid federated learning framework for privacy-preserving near-real-time intrusion detection in IoT environments. Electronics 14:1430. doi: 10.3390/electronics14071430

[ref88] Reji M. Joseph C. Sujatha M. Amanullah M. (2025). A systematic modelling of intrusion detection in IoT using a hybrid machine learning approach. Wirel. Netw 31, 4675–4691. doi: 10.1007/s11276-025-04019-w

[ref89] Rey V. S’anchez P. M. S. Huertas Celdr’an A. Bovet G. (2022). Federated learning for malware detection in IoT devices. Comput. Netw. 204:108693. doi: 10.1016/j.comnet.2021.108693

[ref90] Rishiwal V. Agarwal U. Yadav M. Tanwar S. Garg D. Guizani M. (2025). A new alliance of machine learning and quantum computing: concepts, attacks and challenges in IoT networks. IEEE Internet Things J. 12, 18865–18886. doi: 10.1109/JIOT.2025.3535414

[ref91] Ruiz-Guirola D. E. L’opez O. L. A. Montejo-S’anchez S. (2025). Modeling IoT traffic patterns: insights from a statistical analysis of an MTC dataset. Expert Syst. Appl. 272:126726. doi: 10.1016/j.eswa.2025.126726

[ref92] S’aez-de-C’amara X. . (2023). Clustered federated learning architecture for network anomaly detection in large scale heterogeneous IoT networks. Comput. Secur. 131. doi: 10.1016/j.cose.2023.103299

[ref93] Sasikala S. Janakiraman S. (2023). A review on machine learning-based malware detection techniques for internet of things (IoT) environments. Wirel. Pers. Commun. 132, 1961–1974. doi: 10.1007/s11277-023-10693-w

[ref94] Sathyabama A. R. Katiravan J. (2025). Enhancing anomaly detection and prevention in internet of things (IoT) using deep neural networks and blockchain based cyber security. Sci. Rep. 15:22369. doi: 10.1038/s41598-025-04164-4, 40593899 PMC12215782

[ref95] Selvam P. Karthikeyan P. Manochitra S. Sujith A. V. L. N. Ganesan T. Ayyasamy R. . (2025). Federated learning-based hybrid convolutional recurrent neural network for multi-class intrusion detection in IoT networks. Discov. Internet Things 5. doi: 10.1007/s43926-025-00130-8

[ref96] Shafi M. M. Lashkari A. H. (2025). Toward generating a large-scale IoT-Z wave intrusion detection dataset: smart device profiling, intruders’ behavior and traffic characterization. Internet Things 34:101747. doi: 10.1016/j.iot.2025.101747

[ref97] Shahin M. Hosseinzadeh A. Chen F. F. (2025). A two-stage hybrid federated learning framework for privacy-preserving IoT anomaly detection and classification. Internet Things 6:102685. doi: 10.3390/iot6030048, 30654563

[ref98] Shahin M. Maghanaki M. Hosseinzadeh A. Chen F. F. (2024). Advancing network security in industrial IoT: a deep dive into AI enabled intrusion detection systems. Elsevier. doi: 10.1016/j.aei.2024.102685

[ref99] Sharma S. B. Bairwa A. K., “Leveraging AI for Intrusion Detection in IoT Ecosystems: A Comprehensive Study,” (2025). IEEE: New York, NY, USA.

[ref100] Sharma H. Yadav G. S. (2025). Privacy-preserving intrusion detection with federated learning: enhancing privacy and efficiency in IoT networks. Int. J. Mach. Learn. Cybern. 16, 5821–5839. doi: 10.1007/s13042-025-02599-9

[ref101] Shruti . (2024). Enhancing privacy and security in IoT-based smart grid system using encryption-based fog computing. Alex. Eng. J. 102, 66–74. doi: 10.1016/j.aej.2024.05.085

[ref102] Singh P. R. N. Siddalingaiah N. (2025). Permission-level risk profiling and anomaly detection in IoT using machine learning. Int. J. Inform. Technol. doi: 10.1007/s41870-02502977-0

[ref103] Smmarwar S. K. Gupta G. P. Kumar S. (2023). AI-empowered malware detection system for industrial internet of things. Comput. Electr. Eng. 108:108731. doi: 10.1016/j.compeleceng.2023.108731

[ref104] Sun Y. Wang Z. (2025). Intrusion detection in IoT and wireless networks using image-based neural network classification. Appl. Soft Comput. 177:113236. doi: 10.1016/j.asoc.2025.113236

[ref105] Venkatasubramanian M. Lashkari A. H. Hakak S. (2023). IoT malware analysis using federated learning: a comprehensive survey. IEEE Access 11, 5004–5018. doi: 10.1109/ACCESS.2023.3235389

[ref106] Villafranca A. Cano M. D. (2025). A lightweight edge-DL intrusion detection system for IoT sustainable smart-agriculture. Internet of Thing 34:101818. doi: 10.1016/j.iot.2025.101818

[ref107] Von Der Assen J. A lightweight moving target defense framework for multi-purpose malware affecting IoT devices, in IEEE ICC 2023, pp. 6010–6015.

[ref108] Vyas A. Privacy-Preserving Federated Learning for Intrusion Detection in IoT Environments: A Survey,” (2024). IEEE: New York, NY, USA.

[ref109] Wajahat A. He J. Zhu N. Mahmood T. Nazir A. Ullah F. . (2024). Securing android IoT devices with GuardDroid transparent and lightweight malware detection. Ain Shams Eng. J. 15:102642. doi: 10.1016/j.asej.2024.102642

[ref110] Wakili A. Bakkali S. (2025). Privacy-preserving security of IoT networks: a comparative analysis of methods and applications. Cyber Security Appl. 3:100084. doi: 10.1016/j.csa.2025.100084

[ref111] Wardana A. A. Kołaczek G. Sukarno P. (2024). Lightweight, trust managing and privacy-preserving collaborative intrusion detection for internet of things. Appl. Sci. 14:4109. doi: 10.3390/app14104109

[ref112] Yama Y. Uda R. (2025). “Machine learning approach to malware classification using byte n-grams on IoT devices,” in IMCOM, New York, NY, USA.

[ref113] Yan P. Khoei T., “Securing the Internet of Things: A Comprehensive Review of Ransomware Attacks, Detection, Countermeasures and Future Prospects (2025). 11:100256.

[ref114] Zhukabayeva T. “Enhancing IoT security: effective botnet attack detection through machine learning,” Procedia Computer Science Elsevier B.V. (2024) 421–426

